# Biomarkers of chronic kidney disease in older individuals: navigating complexity in diagnosis

**DOI:** 10.3389/fmed.2024.1397160

**Published:** 2024-07-11

**Authors:** Lucia Muglia, Michele Di Dio, Elvira Filicetti, Giada Ida Greco, Mara Volpentesta, Alessia Beccacece, Paolo Fabbietti, Fabrizia Lattanzio, Andrea Corsonello, Guido Gembillo, Domenico Santoro, Luca Soraci

**Affiliations:** ^1^Centre for Biostatistics and Applied Geriatric Clinical Epidemiology, Italian National Research Center on Aging (IRCCS INRCA), Ancona and Cosenza, Italy; ^2^Unit of Urology, Department of Surgery, Annunziata Hospital, Cosenza, Italy; ^3^Unit of Geriatric Medicine, Italian National Research Center on Aging (IRCCS INRCA), Cosenza, Italy; ^4^Scientific Direction, Italian National Research Center on Aging (IRCCS INRCA), Ancona, Italy; ^5^Department of Pharmacy, Health and Nutritional Sciences, School of Medicine and Digital Technologies, University of Calabria, Arcavacata di Rende, Italy; ^6^Unit of Nephrology and Dialysis, Department of Clinical and Experimental Medicine, University of Messina, Messina, Italy

**Keywords:** older patients, CKD, comprehensive geriatric assessment, chronic kidney disease, frailty, biomarkers

## Abstract

Chronic kidney disease (CKD) in older individuals is a matter of growing concern in the field of public health across the globe. Indeed, prevalence of kidney function impairment increases with advancing age and is often exacerbated by age-induced modifications of kidney function, presence of chronic diseases such as diabetes, hypertension, and cardiovascular disorders, and increased burden related to frailty, cognitive impairment and sarcopenia. Accurate assessment of CKD in older individuals is crucial for timely intervention and management and relies heavily on biomarkers for disease diagnosis and monitoring. However, the interpretation of these biomarkers in older patients may be complex due to interplays between CKD, aging, chronic diseases and geriatric syndromes. Biomarkers such as serum creatinine, estimated glomerular filtration rate (eGFR), and albuminuria can be significantly altered by systemic inflammation, metabolic changes, and medication use commonly seen in this population. To overcome the limitations of traditional biomarkers, several innovative proteins have been investigated as potential, in this review we aimed at consolidating the existing data concerning the geriatric aspects of CKD, describing the challenges and considerations in using traditional and innovative biomarkers to assess CKD in older patients, highlighting the need for integration of the clinical context to improve biomarkers’ accuracy.

## 1 Introduction

Older people represent a very relevant proportion of the population affected by chronic kidney disease (CKD) and end-stage kidney disease (ESKD). In 2017–2020, the prevalence of CKD in older adults aged 65 years or over was 33.2% compared to 9% in adults (1); in 2020 the incidence of ESKD was 1,447 cases per million people among individuals aged 75 or more, 1,225 cases per million people among those aged 65–74 and 598 cases per million people among those aged 45–64 (1).

Older people are more susceptible to CKD for several reasons. Aging can cause anatomical and functional changes in the kidneys, such as the reduction in the number of functioning nephrons and a decrease in renal blood flow (2, 3). Furthermore, some conditions that may contribute to development and progression of CKD, i.e., hypertension, diabetes, cardiovascular disease, and atherosclerosis, are more common in older adults. Furthermore, older people with multimorbidity often take many medications, and some of them, such as non-steroidal anti-inflammatory drugs and some antibiotics can damage the kidneys, especially if taken in high doses or over a prolonged period of time (4–6). Additionally, older people are more susceptible to drug-induced kidney damage due to age-related changes in drug metabolism and excretion (3, 6, 7). Moreover, age-related decrease in renal reserve makes older people more vulnerable to experience kidney damage caused by infections, dehydration, and surgery (3).

To distinguish physiologic aging of the kidney from less severe stages of CKD may be very difficult, which may delay the implementation of specific interventions able to slow the development of CKD complications and ESKD. Additionally, many patients with CKD are asymptomatic or have nonspecific symptoms with the consequence that the course of the disease may remain subclinical for a long time until laboratory clues are searched for. Finally, CKD and its complications may significantly impact functional status and health-related quality of life among older people, and geriatric syndromes (including cognitive status, depression, disability, frailty and sarcopenia) are more and more considered in the assessment of older people with CKD (8).

Therefore, we aimed at summarizing the available evidence about geriatric dimensions of CKD, including the difference between physiological renal ageing and progressive kidney disease, the main issues related to CKD diagnosis and staging, and the challenges related to functional impact of CKD. We will also analyze the potential role of innovative biomarkers in the diagnosis of CKD in older patients, and finally look ahead to the future in this field.

## 2 Ageing kidney and CKD

**Figure 1:**
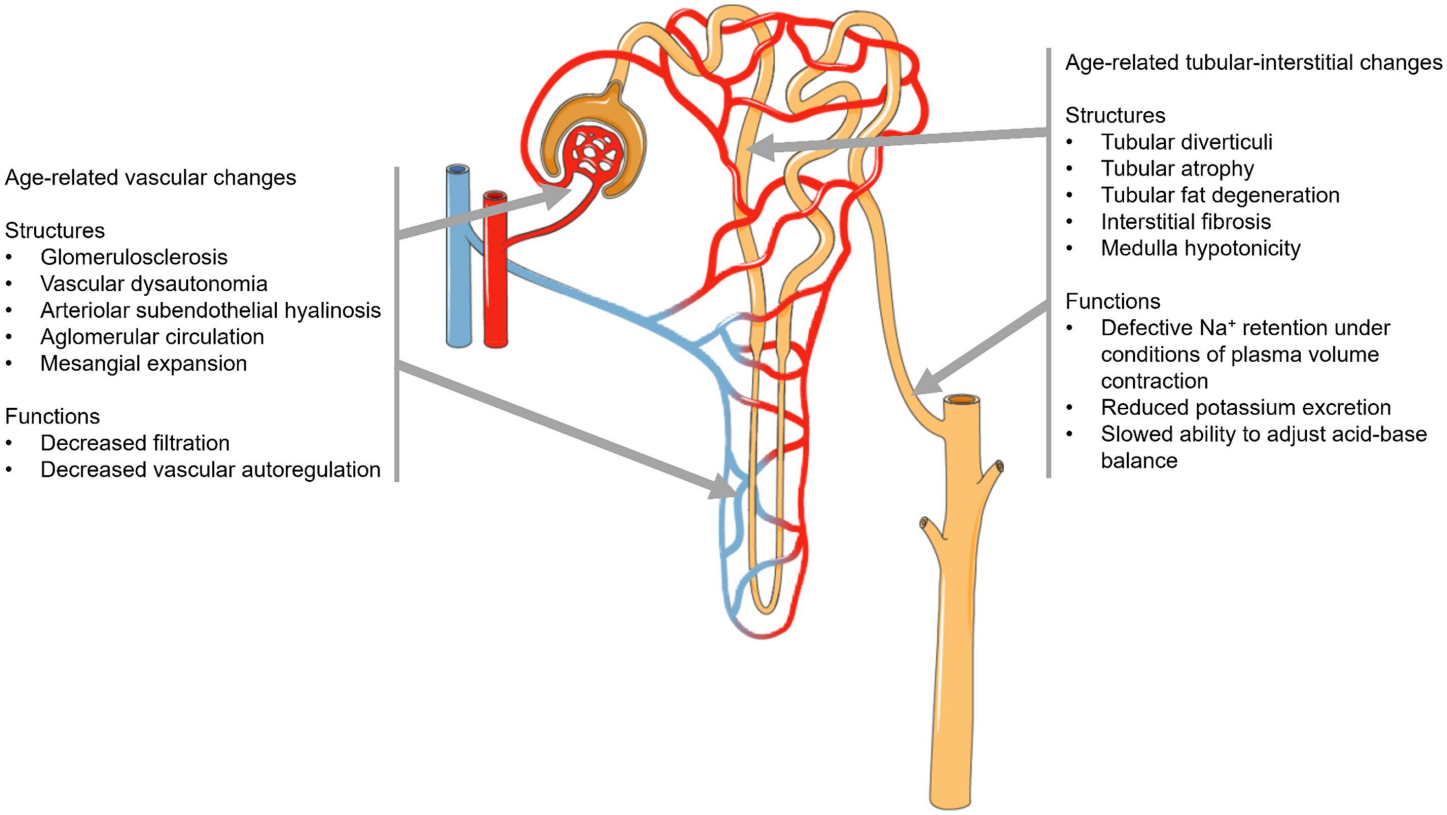
Aging-related changes of kidney structure and function.

Ageing can lead to various changes in kidney structure and function (Figure 1), which may increase the risk of acute kidney injury (AKI) and the development of progressive CKD (9–17). While there are some common pathophysiological and clinical features between the ageing kidney and CKD, the gradual changes that occur with ageing differ from the progressive genetic, immunological, or toxic injury typically associated with CKD (7).

Current evidence suggests that a sort of uneven interplay exists between decreased protective elements (including vascular density, antioxidant capacity, telomere shortening, PPARγ, and Klotho expression) and stress-inducing factors (such as hypoxia, increased expression of collagen I and III, TGF-β, and oxidative stress) associated with renal inflammation and fibrosis, a hallmark of CKD. This imbalance leads to amplified senescence and a reduction in microvascular density, thereby perpetuating damage and driving disease progression (3, 7). Furthermore, advanced glycation end products (AGEs), which accumulate in the bloodstream and tissues with age, could contribute to vascular changes in the kidney and thus to the pathophysiology of both diabetic and non-diabetic CKD. Indeed, AGEs could exacerbate insulin resistance and increase senescence in tubular epithelial cells, affecting renal structures and leading to functional alterations (3, 7).

The main aging-related changes in kidney function may be of clinical significance in older patients, even in the absence of clear evidence of renal disease. Thus, assessment of glomerular filtration rate (GFR) and albumin-to-creatinine ratio (ACR) together with the assessment of the presence of other systemic diseases or clinical conditions (e.g., cardiovascular and respiratory diseases, diabetes, muscle-skeletal disorders, and polypharmacy) can help clinicians interpret the clinical relevance of age-related changes in renal function (7).

## 3 Biomarkers of kidney function

### 3.1 Traditional biomarkers of kidney function

CKD is typically identified by the presence of a GFR below 60 mL/min/1.73 m^2^ or albuminuria of at least 30 mg per day, associated with the presence of urinary and serum biomarkers of renal damage. According to Kidney Disease: Improving Global Outcomes (KDIGO) clinical practice guidelines, CKD is defined as a GFR below 60 mL/min/1.73 m^2^ or the presence of kidney damage, such as albuminuria ≥ 30 mg/day, for a duration of at least 3 months (8).

#### 3.1.1 Serum creatinine and eGFR

In clinical practice, due to the complexity and limited applicability of gold-standard methods for measuring GFR in a clinical setting, the assessment of eGFR is commonly carried out by using simple creatinine-based equations (18). This approach has partly overcome the problems related to measurement of creatinine only, which is still the most commonly used endogenous marker of glomerular function (19). Creatinine is produced in the muscles as a by-product of the breakdown of creatine phosphate, which the body makes up in a relatively constant ratio to muscle mass; creatinine is mainly excreted by the kidneys, where it is freely filtered through the glomerulus and released mainly via organic transporters in the proximal tubules. Assessment of serum creatinine can raise some issues in older individuals, as its serum levels can fluctuate in relation to muscle mass and function, body composition, exercise, and diet (19). Moreover, its excretion may increase in individuals with severe albuminuria, e.g., those with nephrotic syndrome, resulting in false negative readings (20). Consequently, changes in creatinine levels do not always align with parallel changes in renal function (5, 21); to address the limitations of serum creatinine assessment, in last decades several age- and sex-adjusted creatinine-based equations to estimate GFR have been developed. With this aim, the Chronic Kidney Disease Epidemiology Collaboration (CKD-EPI) has developed an estimating equation that is more accurate than creatinine clearance alone and was internally validated in 10 different CKD cohorts using urinary iothalamate clearance to quantify GFR (22). Years after the development of this formula, the newly created CKD-EPI 2021 equation has been shown to be more accurate by reducing the overestimation of GFR in black patients (23). However, accuracy of CKD-EPI equation in older individuals is still matter of debate; indeed, numerous studies have demonstrated a U-shaped relationship between creatinine-based eGFR and mortality in older individuals, meaning that high risk of mortality was shared by older patients with eGFR considered to be normal (24, 25). This is thought to be due to the muscle mass depletion which is relatively common in geriatric population and can lead to overestimation of creatinine and consequently eGFR (26). In this regard, the introduction of two eGFR equations specifically designed in older cohorts, namely the Berlin initiative study (BIS) (27) and the full age spectrum (FAS) equation (28) has represented a new frontier in diagnosis of CKD in geriatrics. Several studies have shown that BIS/FAS equations improved both eGFR estimation and prognostic risk stratification in older individuals (29–32). In contrast to CKD-EPI equation, specifically designed for estimating GFR in the adult population, the BIS equation considers age-related changes of muscle mass for more accurate GFR estimates among older individuals; in contrast, the FAS equation applies to all ages, making it versatile for diagnosis and managing CKD across the lifespan (30). However, results are conflicting and effects of sarcopenia and other nonrenal factors on serum creatinine are not adjusted in BIS and FAS equations (33); for this reason, cystatin C (CysC), which is known to be less affected by muscle turnover than serum creatinine (34), is emerging as a biomarker to estimate renal function in the older with sarcopenia and frailty (35–37).

#### 3.1.2 CysC

CysC is a small protein (12 kDa) that is synthesized in all cells of the body containing a nucleus, at a consistent pace. It undergoes unimpeded filtration by the glomerulus with complete tubular reabsorption and catabolization, devoid of reabsorption into the bloodstream and renal tubular secretion. For this reason, CysC is considered a reliable indicator of filtration (38). Research has indicated that CysC levels increase more rapidly than serum creatinine levels following AKI (39). Equations based on CysC for estimating glomerular filtration rate (GFR) (or a combination of CysC and sCr equations) have been shown to be superior to equations based solely on creatinine (40–42). Unlike creatinine, CysC is less influenced by muscle mass and gender, and cystatin C-based equations have been shown to improve prediction of adverse health outcomes in CKD older patients compared with creatinine-based ones (43–46). However, the improvement was generally slight, and the higher cost of CysC compared to creatinine assessment hinders CysC implementation in clinical practice. Indeed, it has been suggested that CysC might represent a cost-effective option to estimate renal function in young adults only, where it has the potential to decrease the rate of false positives but not among older individuals (47). However, use of CysC may be proposed in selected subgroups of older populations, especially those with sarcopenia and physical frailty (48), where it could be a more reliable and muscle-mass-independent marker of renal function.

#### 3.1.3 uACR

Urinary albumin-to-creatinine ratio (uACR) is the recommended measure for assessing albuminuria and has also been used to predict the risk of renal failure in CKD patients (49, 50) and it was included in the CKD staging system as well as in risk scores to predict the occurrence of renal failure (50). Albuminuria tests are often less prone to analytical inaccuracies than total protein tests, especially at lower proteinuria levels. In addition, albumin is the most common protein in urine in a variety of kidney diseases, and urinary albumin levels can be accurately measured within the normal range (51). For these reasons, guidelines recommend the uACR as the best test to assess albuminuria/proteinuria (52, 53). Additionally, ACR can accurately predict cardiovascular endpoints and other endpoints, along with the risk of developing CKD among individuals with high-to-normal eGFR (54–57). Furthermore, a recent meta-analysis demonstrated that elevated uACR levels are independently associated with an augmented risk of hypertension (58). In high-risk patients, such as those with diabetes mellitus, regular uACR assessment is recommended to reliably classify risk of cardiovascular disease and all-cause death (57, 59, 60). However, ACR determination presents also some limitations, mainly regarding its fluctuations caused by some nonrenal factors such as physical exercise, fever, and infections, that may make its evaluation less reliable in the acute settings (61).

The mentioned limitations can subsequently hinder clinicians’ ability to accurately identify individuals at risk of CKD and forecast their prognosis when treatments can potentially mitigate future risks, as recommended in guidelines (62). Considering this, novel biomarkers are receiving increasing attention to enhance the prognostic accuracy and surveillance in CKD older patients. Therefore, it is imperative and advantageous to explore sensitive diagnostic measures and novel biomarkers that are receiving increasing attention to enhance the diagnostic and prognostic efficiency and surveillance of CKD (63).

### 3.2 Novel biomarkers of kidney function

**Figure 2:**
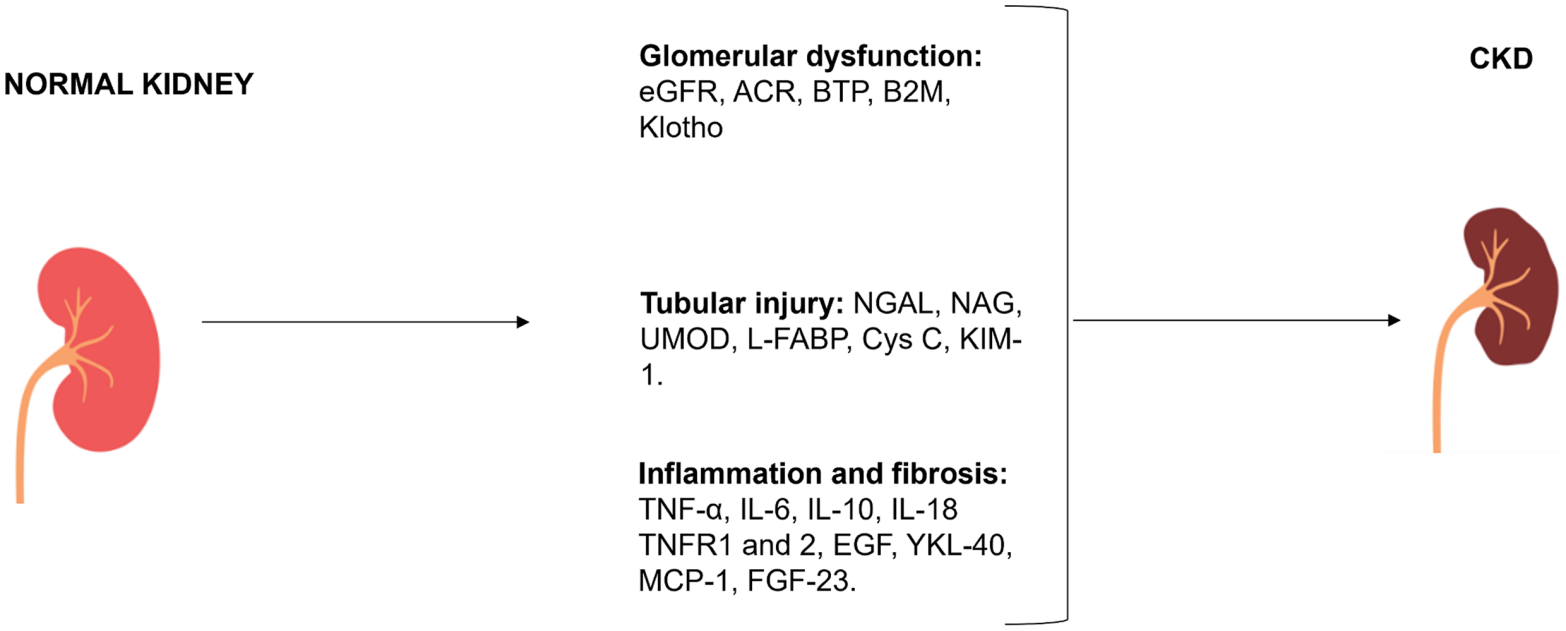
Classification of plasma and urinary CKD biomarkers according to mechanism of action.

The evaluation of renal function is predominantly dependent on the estimation of albuminuria and GFR, which is achieved through the utilization of equations based on creatinine or both creatinine and ACR. Considering the acknowledged limitations associated with these markers, numerous alternative markers have been extensively investigated to enhance the accuracy of renal function assessment. Some biomarkers were also capable of predicting adverse outcomes (e.g., AKI incidence, CKD progression, cardiovascular events, and death) and may be used as prognostic indicators in CKD patients. The classification of main traditional and novel biomarkers involved in CKD is reported in Figure 2 and in Tables 1–3.

**Table 1:** Summary of findings of longitudinal studies investigating the association between biomarkers of glomerular function and outcomes in older patients with CKD.

Study	Population	Outcomes	Main findings
Rebholz et al. 2015 (prospective cohort)	9,703 participants of the ARIC study with a mean age of 58 years Follow-up time of 6 years	Incidence of ESKD	A decline of at least 30% of B2M-based eGFR improve specificity in predicting ESKD compared to creatinine-based eGFR
Foster et al. 2016 (prospective cohort)	3,613 adults of the CRIC study with a mean age of 58 years Follow-up time of 6 years	Incidence of CKD, all-cause mortality and cardiovascular events	BTP and B2M were associated with all-cause mortality and ESKD, while only B2M predicted cardiovascular events
Drew et al. 2017 (prospective cohort)	2,496 older adults of the Health-ABC study with a mean age of 70 years	eGFR decline and CKD incidence	Lower soluble Klotho levels were associated with eGFR decline
Rebholz et al. 2017 (prospective cohort)	2 cohorts: 317 patients aged 52 years of the MDRD study; 373 patients aged 56 years of the AASK study Follow-up time of 5 and 7 years	Incidence of ESKD and all-cause mortality	1-year decline of eGFR based on BTP was associated with ESKD in both cohorts; this association was stronger than that between mGFR and study outcome
Inker et al. 2017 (meta-analysis)	6 cohorts: 4 of them (higher risk cohort had a mean age of 60 years) Mean follow-up time of 6 years for ESKD and 14 years for mortality	ESKD and all-cause mortality	eGFR_BTP_ and eGFR_cys_ improved prediction of ESKD and mortality compared with eGFR_cr_
Qian et al. 2018 (prospective cohort)	112 patients with stage 1–5 CKD with a mean age of 64 years Mean follow-up time of 6 years	Start of renal replacement therapy and cardio-cerebrovascular events	Decline in Klotho levels over time was associated with eGFR decline
Liu et al. 2019 (meta-analysis)	Meta-analysis of 8 studies including 3,586 patients	Annual eGFR decline, risk of renal replacement therapy	Low Klotho levels were associated with study outcomes
Memmos et al. 2019 (prospective cohort)	79 patients on maintenance dialysis with a mean age of 60 years Median follow-up time of 5.5 years	All-cause mortality, CV mortality, composite outcome	Low Klotho levels were associated with study outcomes
Silva et al. 2019 (prospective cohort)	107 patients with diabetes and a mean age of 59 years Mean follow-up time of 34 months	Cardiovascular risk and hospitalization	Low Klotho levels were associated with cardiac structural changes and cardiovascular risk
Leyssens et al. 2021 (prospective cohort)	52 intensive care unit patients with a mean age of 64 years	AKI	BTP had a higher discriminative ability for AKI prediction than NGAL
Buyadaa et al. 2023 (prospective cohort)	1,604 patients with diabetes and CKD with a mean age of 61 years	Annual rate of eGFR decline	Low serum BTP was associated with normoalbuminuria and slower eGFR decline

AKI, acute kidney injury; ARIC, Atherosclerosis Risk in Communities; B2M, beta-2-microglobulin; BTP, beta-trace protein; CV, cardiovascular; eGFR, estimated glomerular filtration rate; ESKD, end-stage kidney disease; Health-ABC, Health Aging and Body Composition; MACE, major adverse cardiovascular events; MDRD, Modification of Diet in Renal Disease; NGAL, neutrophil gelatinase-associated lipocalin; OPN, osteopontin.

**Table 2:** Longitudinal studies showing the associations between plasma and urinary tubular biomarkers and outcomes in older patients with CKD.

Study	Population	Outcomes	Main findings
Lim et al. 2015 (prospective cohort)	Women >70 years Follow-up: 5 years for eGFR. 10 years for hospitalization and mortality	5-year eGFR change, acute eGFR decline, 10-year risk of hospitalization and mortality	Plasma NGAL was associated with increased risk of rapid renal decline and 10-year risk of renal disease events, especially in mild-moderate CKD
Jungbauer et al. 2015 (prospective cohort)	138 patients with heart failure and a mean age of 62 years Follow-up: 5 years	CKD progression All-cause mortality	Urinary KIM-1 and NAG, but not NGAL, were associated with CKD progression and mortality
Alderson et al. 2016 (prospective cohort study)	1982 older adults with a mean age of 64 years	ESKD, all-cause mortality and cardiovascular events Median follow-up time of 29.5 years	In moderate-severe CKD stages, plasma NGAL and KIM-1 predicted the incidence of ESKD and all-cause mortality
Hasegawa et al. 2016 (prospective cohort)	252 patients with CKD and a mean age of 67 years	Cardiovascular events Median follow-up time of 63 months	Plasma NGAL is an independent predictor of cardiovascular events
Matsui et al. 2016 (prospective cohort)	244 Japanese outpatients with CKD and a median age of 64 years	Fatal or nonfatal CVD event CKD progression Median follow-up time of 3.8 years	High urinary L-FABP and low eGFR were associated with incidence of ESKD and CVD events, irrespective of diabetes
Khatir et al. 2017 (prospective cohort)	74 patients with stage 3–4 CKD and a mean age of 61 years	eGFR change Follow-up time of 18 months	Urinary L-FAPB/Creatinine was associated with eGFR change
Moriya et al. 2017 (prospective cohort)	102 healthy patients with a mean age of 59 years; 112 patients with CKD and a mean age of 66 years	eGFR changes Follow-up time of 18 months	Plasma NGAL was associated with eGFR decline in patients with early-stage CKD
Lobato et al. 2017 (prospective cohort)	250 patients with CKD and a mean age of 59 years	CKD progression to ESKD, mortality Median follow-up time of 15 months	Urinary NGAL was moderately correlated with stage 5 CKD and incidence of ESKD; associations were very mild for KIM-1 and NAG
Zhang et al. 2018 (case-control study)	324 participants of the SPRINT trial with a mean age of 64 years	Incidence of CKD Follow-up time of 1 year	Urinary KIM-1 but neither NGAL nor uromodulin was associated with incidence of CKD
Seibert et al. 2018 (prospective cohort)	143 older patients with stable CKD and a median age of 72 years	CKD progression Median follow-up time of 3 years	Neither urinary NGAL, nor KIM-1 nor calprotectin were associated with CKD progression
Żyłka et al. 2018 (retrospective cohort)	80 patients with type 2 diabetes and a mean age of 59 years Follow-up time of 1 years (sub-cohort of 29 patients)	eGFR decline uACR worsening	Serum and urinary NGAL were associated with eGFR decline Urinary NGAL and KIM-1 were associated with uACR worsening
Steubl et al. 2019 (prospective cohort)	230 patients with stage 1–4 CKD and a mean age of 60 years	Incidence of ESKD and 25% eGFR decline Mean follow-up time: 57 months	Urinary uromodulin concentrations were associated with CKD progression
Steubl et al. 2019 (case-control study)	933 older patients with a mean age of 78 years	ESKD Follow-up time of 10 years	Lower serum uromodulin levels were associated with ESKD, independent of eGFR, uACR and cardiorenal covariates
Garimella et al. 2019 (prospective cohort)	2,377 older patients of the SPRINT trial with nondiabetic CKD and a mean age of 73 years	MACE+CV death All-cause mortality Median follow-up time of 3.8 years	Lower urinary uromodulin and higher urinary alfa-1-microglobulin levels were associated with increased risk of all study outcomes
Malhotra et al. 2020 (prospective cohort)	2,428 SPRINT participants with CKD and a mean age of 73 years	eGFR decline, progression to ESKD Median follow-up time of 3.8 years	Urinary KIM-1, MCP-1, YKL-40, and IL-18 were associated with study outcomes
Schulz et al. 2020 (prospective cohort)	4,739 older adults with a mean age of 57 years	CKD incidence and eGFR decline Mean follow-up time of 17 years	Plasma KIM-1 levels predict renal outcomes in healthy individuals
Chen et al. 2022 (prospective cohort)	1,135 older Veterans with albuminuric CKD, diabetes and a mean age of 65 years	eGFR decline, all-cause mortality Median follow-up time of 2 years	Urinary MCP-1 and YKL-40 were associated with eGFR decline Urinary MCP-1, YKL-40, NGAL and KIM-1 were associated with increased mortality
Puthumana et al. 2021 (prospective cohort)	1,538 hospitalized older patients with a mean age of 65 years	eGFR decline, CKD incidence, progression, ESKD, all-cause mortality Median follow-up time of 4.3 years	High urinary MCP-1 and YKL-40 were associated with faster eGFR decline, CKD progression and all-cause mortality; higher urinary UMOD was associated with smaller eGFR decline
Buyadaa et al. 2023 (prospective cohort study)	1,604 older adults from the CRIC study with a mean age of 60 years	CKD progression Normoalbuminuria Follow-up of more than 10 years	Low serum KIM-1 was associated with slow eGFR decline
Vasquez-Rios et al. 2023 (prospective cohort)	560 patients with diabetes and CKD and with a mean age of 70 years	All-cause and disease-specific mortality Mean follow-up time of 6 years	Higher urine KIM-1 and YKL-40 were associated with increased risk of all-cause mortality Higher urine UMOD and MCP-1 were associated with increased cardiovascular and cancer-related death

CKD, chronic kidney disease; CV, cardiovascular; KIM, kidney injury molecule; L-FABP, liver-type fatty acid binding protein; MACE, major adverse cardiovascular events; MCP, monocyte chemotactic protein; NAG, N-acetyl-β-D-glucosaminidase; NGAL, neutrophil gelatinase-associated lipocalin; UMOD, uromodulin; YKL-40, chitinase-3-like protein 1.

**Table 3:** Longitudinal studies showing the associations between plasma and urinary markers of inflammation and outcomes in older patients with CKD.

Study	Population	Outcomes	Biomarkers	Main findings
Amdur et al. 2016 (prospective cohort)	3,430 patients with decreased eGFR and with a mean age of 59 years	eGFR decline, ESKD Median follow-up time of 6.3 years	Plasma TNF-α and IL-6	TNF-α and IL-6 were associated with study outcomes
Sjöberg et al. 2016 (prospective cohort)	1,419 older patients with a mean age of 76 years	Incident CKD Follow-up time of 5 years	Serum PTX3	PTX 3 was associated with incident CKD
Sun et al. 2016 (prospective cohort)	543 patients on dialysis and a mean age of 56 years	All-cause and CV mortality Follow-up time of 5 years	Plasma IL-6, TNF-α, WBC, troponin T, IGF-1, orosomucoid, hsCRP, sVCAM-1	IL-6, WBC and TNF-α were independent predictors of the outcome; only low TNF-α and high WBC predicted CV mortality
Fernàndez-Juàrez et al. 2017 (RCT)	101 patients with type 2 DKD and a mean age of 70 years	Composite (eGFR decline, ESKD or death) Median follow-up time of 32 months	Serum TNFR1 and 2	TNFR1 was associated with increased CKD progression and mortality
Gohda et al. 2017 (prospective cohort)	319 patients undergoing hemodialysis with a mean age of 66 years	All-cause and CV mortality Median follow-up time of 53 months	Serum TNFR1 and 2	TNFR1 and 2 were strongly associated with increased mortality
Nair et al. 2017 (prospective cohort)	521 older patients with CKD and a mean age of 60 years	30% eGFR decline, progression to ESKD Follow-up time of 2 years	Serum or plasma GDF-15	Circulating GDF levels predicted study outcomes
Krzanowski et al. 2017 (prospective cohort)	78 patients with stage 5 CKD and a mean age of 61 years	All-cause and CV mortality Follow-up time of 5 years	IL-6, 18, hsCRP, TNFR2, TGF-β_1_, HGF, TM, SDF-1α, osteocalcin, osteoprotegerin, osteopontin	Only PTX3 levels predicted all-cause mortality; both hs-CRP and PTX3 predicted CV mortality, but PTX3 only improved risk stratification compared with CV risk factors
Tuegel et al. 2018 (prospective cohort)	883 older adults with CKD and a mean age of 57 years	All-cause mortality, CV events Follow-up time of 8 years	Serum GDF-15, Gal-3, sST2	GDF-15, Gal-3, sST2 predicted all-cause mortality; GDF-15 only predicted incidence of heart failure
Frimodt-Møller et al. 2018 (prospective cohort)	200 patients with type 2 diabetes and a mean age of 59 years	eGFR decline, CV events and all-cause mortality Median follow-up time of 6.3 years	Plasma GDF-15 and FGF-23	High plasma GDF-15 predicted incidence of eGFR decline; in patients with microalbuminuria, GDF-15 predicted all-cause mortality
Bansal et al. 2019 (prospective cohort)	3,664 older adults with CKD and a mean age of 57 years	CKD progression Median follow-up time of 5.7 years	Plasma GDF-15, sST-2, hs-troponin T and NT-proBNP	GDF-15 and NT-proBNP were associated with increased risk of CKD progression
Kamińska et al. 2019 (prospective cohort)	57 older adults with stage 3–5 CKD and a mean age of 60 years	All-cause mortality Follow-up time of 5 years	Serum IL-6, TNF-α, VCAM-1, ICAM-1, fetuin A, adiponectin, leptin, MMP-9	IL-6 was correlated with calcium score and was associated with mortality
Valente et al. 2019 (prospective cohort)	246 patients with ESKD and a median age of 71 years	All-cause mortality Follow-up time of 1 year	Plasma PTX3, IL-6, TNF- α, CRP, NT-proBNP, TIMP-1	PTX3 only was associated with mortality
Li et al. 2020 (prospective cohort)	160 patients with DKD and a mean age of 62 years	eGFR change Follow-up time of 3 years	Serum *cf*-DNA	It was associated with eGFR decline at 1.5 and 3 years
Batra et al. 2021 (prospective cohort)	14,611 patients with chronic coronary syndrome and mean age of 65 years	MACE, all-cause and CV mortality Median follow-up time of 3.6 years	Serum IL-6, NT-proBNP, hs-CRP, troponin T	Higher IL-6 levels were associated with all-cause, CV mortality and MACE
Scurt et al. 2021 (RCT)	360 older adults with nonalbuminuria and diabetes from the ROADMAP trial with a median age of 58 years	Incidence of microalbuminuria Median follow-up of 6.5 years	Serum and urine MCP-1	Both were associated with study outcome
Waijer et al. 2022 (post-hoc analysis of RCT)	3,532 older adults with type 2 diabetes from the CANVAS study and a mean age of 63 years	Renal outcome (eGFR decline, kidney failure, ESKD) Hospitalization for heart failure Median follow-up time of 6.1 years	Plasma TNFR1, TNFR2 and KIM-1	TNFR1 and 2 detected patients with albuminuria at risk of CKD progression
Li et al. 2023 (prospective cohort)	428 incident dialysis patients with a median age of 56 years	All-cause and CV mortality Follow-up time of 5 years	Plasma IL-6 and albumin	IL-6/albumin ratio was associated with study outcomes

cf-DNA, cell-free DNA; CV, cardiovascular; Gal 3, galactin 3; GDF-15, growth differentiation factor 15; FGF-23, fibroblast growth factor-23; HGF, hepatocyte growth factor; hs-CRP, high-sensitivity C-reactive protein; ICAM-1, intracellular-1 adhesion molecule; IGF, insulin-like growth factor 1; interleukin; KIM-1, kidney injury molecule-1; MCP-1, monocyte chemoattractant protein-1; MMP-9, metalloproteinase 9; NGAL, neutrophil gelatinase-associated lipocalin; NT-proBNP, N-terminal pro-B-type natriuretic peptide; PTX3, pentraxin 3; SDF-1α, stromal cell-derived factor 1α; sST2, soluble ST2; sVCAM-1, Soluble Vascular Cell Adhesion Molecule-1; TIMP-1, tissue inhibitor of metalloproteinase-1; TNF-α, tumor necrosis factor α; VCAM-1, vascular-1 adhesion molecule; YKL-40, chitinase-3-like protein 1.

#### 3.2.1 Markers of glomerular function

Evidence on markers of glomerular function mainly comes from longitudinal studies conducted in middle aged and older adults, while only a minority has been specifically conducted in geriatric populations. A summary of these studies is reported in Table 1 (64–75).

##### 3.2.1.1 BTP and B2M

Beta trace protein (BTP) and β2-microglobulin (B2M) are emerging as novel biomarkers of kidney function, as they are filtrated by glomeruli and almost entirely reabsorbed by the proximal tubules (76, 77). Therefore, increase in serum BTP and B2M concentration is a potential indicator of decreased GFR (76–78), with the advantage that these biomarkers appear to be less influenced by age, sex and race compared with serum creatinine (76, 77); furthermore, as these proteins undergo proximal tubule reabsorption, increase in their urinary concentration indicate tubular damage (78).

BTP is a protein catalyst of the conversion of prostaglandin H2 into prostaglandin D2. Previous studies have shown that increased urinary and plasma BTP concentrations were highly correlated with serum levels of creatinine and cystatin C. Many studies have compared its diagnostic efficacy with conventional CKD biomarkers, such as creatinine, cystatin C and ACR, disclosing that increases in serum and urinary BTP levels correlate strongly with creatinine and cystatin C (79–81). Although BTP has a lower accuracy in eGFR estimation than cystatin C (73, 82–85), it appears to be less influenced by race (86) and its assessment has been suggested particularly in cases where creatinine does not provide accurate results (e.g., in the creatinine-blind range) (85, 87); in this context, lower serum BTP has been recently associated with slower GFR decline in older patients with normoalbuminuric CKD and diabetes (74). BTP was also associated with ACR and may be an early indicator of diabetic kidney disease (DKD); CKD patients with type 2 diabetes and microalbuminuria were found to have higher serum and urinary BTP levels than patients with normalbuminuria (88); furthermore, eGFR_BTP_ improved prediction of CKD progression to ESKD and mortality compared to traditional eGFR measurements (66, 67).

B2M is a major histocompatibility class I molecule produced by most nucleated cells and present in many biological fluids (89). Its serum and urine concentrations tend to increase with decrease in eGFR (90, 91), but may be also affected by some non-renal conditions, such as infectious diseases, cancer, and aging (92). Like BTP, the role of B2M in diabetes has been extensively studied; a recent meta-analysis of 8 cohort studies has shown that B2M levels are associated with increased risk of DKD (93). Furthermore, a decrease in eGFR of at least 30% based on B2M determination has been shown to strongly predict the incidence of ESKD (75). Furthermore, in adults with CKD from the Chronic Renal Insufficiency Cohort (CRIC), both B2M and BTP predicted all-cause mortality, but B2M was specifically associated with cardiovascular mortality (64); however, the study population included individuals aged 25–75 years, thus not fully representative of the geriatric population. Finally, studies comparing the accuracy of the two biomarkers in predicting kidney disease have produced rather contradictory results. In some studies, there was no advantage in terms of predictive accuracy when BTP and B2M were included in the traditional eGFRcys and eGFRcr equations (83, 94). On the other hand, a recent meta-analysis comprising six studies has shown that eGFR based on BTP and B2M improves the detection of patients at risk of ESKD compared to the traditional eGFR equations (67). Furthermore, a recent cross-sectional analysis showed that estimation of eGFR by use of CKD-EPI_BTP-B2M_ was independently associated with sarcopenia in community-dwelling older individuals in contrast to creatinine and cystatin C-based equations (95).

##### 3.2.1.2 Klotho

Klotho is a novel antiaging transmembrane protein expressed in proximal and distal tubule cells and its concentration is directly proportional to renal function (96). For this reason, low concentrations of soluble α-Klotho are considered a sensitive and early marker of CKD, even in stage 1 and 2 patients (97, 98). In addition, changes in soluble Klotho could serve as an indicator of CKD progression, as they correlate with changes in eGFR over time (69, 99). In a recent prospective cohort study, circulating α-Klotho levels have been associated with increased risk of AKI, CKD progression and death, even after adjusting for fibroblast growth factor 23 (FGF-23) levels (100). This result was also confirmed in a small sample of 79 patients undergoing hemodialysis, where low Klotho resulted to independently predict AKI incidence, CKD progression, and survival (100). A progressive decrease in soluble Klotho levels with decreasing eGFR could contribute to cardiovascular and cerebrovascular changes and increase the risk of cardiovascular mortality and adverse events.

In patients with CKD, a reduction in Klotho concentrations is observed and has been found associated with increased albumin excretion, a higher risk of cardiovascular disease (CVD), mortality, and inflammation. In addition, Klotho acts as the co-receptor for FGF-23 (101) and appears to play a role in the pathophysiology of ion disturbances, contributes to regulation of bone calcium and phosphorus metabolism (102) and to the prevention of renal fibrosis (101, 103). A study conducted on 125 patients undergoing dialysis has shown that serum Klotho levels strongly correlated with the severity of mineral bone disorder (104). Furthermore, Klotho levels negatively correlated with serum phosphatemia, indicating that a decrease in Klotho levels may exacerbate urinary phosphate excretion disorders (105). Furthermore, given its correlations with muscle mass and quality, Klotho may represent the biomolecular link between sarcopenia and CKD (106).

#### 3.2.2 Markers of tubular function

Tubular function can be impaired at early stages of CKD, even in the absence of overt glomerular dysfunction. Therefore, increase in serum and urinary tubular biomarkers may represent a future option to capture prognostic risk in patients with early CKD (107). In addition, they can be used as safety biomarkers for monitoring nephrotoxicity in clinical trials (108). Among most interesting biomarkers of tubular function, we mention neutrophil gelatinase-associated lipocalin (NGAL), kidney injury molecule-1 (KIM-1), N-acetyl-β-D-glucosaminidase (NAG), liver-type fatty acid binding protein (L-FABP), uromodulin (UMOD). Evidence from clinical longitudinal studies on tubular markers and CKD in middle aged and older individuals is summarized in Table 2 (70, 74, 109–126).

##### 3.2.2.1 NGAL

Lipocalin 2 (LCN2) or NGAL is one of the most promising and investigated biomarkers of kidney disease. This glycoprotein is associated with matrix metalloproteinase-9 in human neutrophils and is relevant in the transport of hydrophilic compounds between cells and in defense against microbes (127). Both plasma and urinary NGAL were associated with increased risk of acute kidney injury following cardiac surgery (128); compared with plasma NGAL, the urinary metabolite appears to be more specific of kidney injury, which determines NGAL gene up-regulation at the level of distal nephron segments, thus increasing is urinary concentration (129). However, evidence from clinical studies draw contrasting results. Indeed, on one hand plasma NGAL has been associated with uACR (130) and had modest ability to predict renal function decline and AKI in older women (109); in selected older individuals with type I diabetes, plasma NGAL levels were associated with increased risk of DKD over a 12-year follow-up (130); similarly, plasma NGAL has been associated with eGFR changes in patients with early stage CKD (115); furthermore, in a large cohort of 1982 older adults with moderate-severe CKD, both plasma NGAL and Kim-1 predicted the incidence of ESKD, as well as all-cause mortality (111). On the other hand, urinary NGAL presented an inverse relationship with the eGFR and a direct correlation with both interstitial fibrosis and tubular atrophy (131). In one study on patients with CKD, urinary NGAL was moderately correlated with stage 5 CKD and predicted the incidence of ESKD (116); moreover, in patients with DKD, both serum and urine NGAL were associated with eGFR decline, while urine NGAL with uACR worsening over time (119). As regards the association with cardiovascular events, both plasma and urinary NGAL have been associated with incidence of cardiovascular events among patients with CKD (112, 132). However, other studies showed negative or no association between NGAL and renal or cardiovascular outcomes (110, 116–118).

##### 3.2.2.2 NAG and KIM-1

N-acetyl-β-D-glucosaminidase (NAG) and kidney injury molecule (KIM-1) have recently been proposed as early CKD biomarkers (133, 134).

NAG is a glycosidase of high molecular weight and mainly contained in the lysosomes of proximal tubular cells. As it cannot be filtered by glomerular cells, its urinary concentrations are strongly related with proximal tubule damage (135).

KIM-1, also known as T cell immunoglobulin or mucin-containing molecule, is a cellular receptor involved in immune function regulation and response to viral infections (136); it is expressed in proximal tubular cells in response to damage and is involved in promoting renal fibrosis; additionally, it has been found to be a potential marker of glomerular function, because of its increased expression in diabetic glomerulopathy. For these reasons, it is considered a very sensitive and specific marker of kidney injury, and it is upregulated in AKI and CKD (137) after toxic and ischemic injury as it contributes to renal repair (138). Indeed, chronic stimulation of KIM-1 production induces up-regulation of monocyte chemotactic protein 1 (MCP1) which in turn induces proinflammatory responses and renal fibrosis (139). Interestingly, plasma KIM-1 can predict the progression of CKD to ESKD (111) and the progression to CKD in patients with diabetes (137).

Studies investigating the role of NAG and KIM-1 on cardiorenal outcomes in CKD led to contrasting results. Indeed, in a large cohort a small cohort of older patients with heart failure, both urinary biomarkers were associated with CKD progression over a 5-year follow-up time (110). On the other hand, in another cohort of older patients with CKD, both urinary NAG and KIM-1 were not associated with CKD progression and ESKD, in contrast with NGAL (116); evidence of the prognostic potential of urinary KIM-1 stemmed from two studies derived from the SPRINT trial; in the first one, including a sub-cohort of 328 older adults with hypertension, urinary KIM-1 levels predicted the incidence of CKD among patients with type 2 diabetes and normal baseline renal function (117); in another cohort of over 2000 patients from the same trial, patients with highest KIM-1 and NGAL quartiles were at risk of increased CKD progression (122). Furthermore, in a small cohort of 80 patients with a mean age of 59 years, urinary KIM-1/creatinine was associated with increased uACR at 1 year (119).

##### 3.2.2.3 L-FABP

Liver-type fatty acid-binding protein (LFBP) is mainly produced by liver and proximal tubular cells (140). This protein exhibits a specific affinity for free fatty acids and facilitates their transportation to either mitochondria or peroxisomes (141). The circulating form of L-FABP is believed to undergo filtration at the glomeruli and subsequent reabsorption by proximal tubular cells and is expressed in the proximal tubules of humans following acute ischemic injury (141). Consequently, increased levels of L-FABP have been established as a sensitive and specific biomarker for tubulointerstitial damage AKI in both adult and older populations (142, 143). Despite being expressed in the liver, previous studies have shown that urinary L-FABP levels are not considerably elevated in patients with liver disease (143, 144). Potential usefulness of urinary L-FABP stems from clinical evidence showing its negative correlation with eGFR and renal function decline (114); such evidence was confirmed also in prospective cohorts of CKD older patients, where high urinary L-FABP has been associated with eGFR decline in patients without albuminuria (114), as well as with progression to ESKD and incidence of CVD, irrespective of diabetes (113). Similarly, Matsui et al. (113) showed that high urinary L-FABP levels were associated with increased risk of cerebrovascular disease and ESKD in a cohort of Japanese older adults; some correlation with electrocardiographic features and troponin elevation may explain these associations (145).

##### 3.2.2.4 UMOD

Uromodulin (UMOD), also named as Tamm–Horsfall protein, is a glycoprotein exclusively synthesized by the cells of the thick ascending limb of Henle (146); it is implicated in the regulation of salt homeostasis and the bestowal of immunological protection to the kidneys, thus contributing to defense against infective diseases and kidney stones (147). It is one of the most abundant components of normal urine (146) and a potential biomarker of tubular function. However, a small part of urinary UMOD can be secreted in the circulation, with a serum concentration much more lower than urinary UMOD (148). Recent studies have shown that serum UMOD levels exhibit positive correlations with eGFR, being lower in patients with CKD (149), while higher levels were associated with structure integrity of the renal parenchyma (148). Furthermore, Steubl et al. (120, 121) showed that lower serum UMOD levels were associated with ESKD, independent of eGFR and uACR, while lower urinary UMOD was associated with CKD progression in two cohorts of older adults. Garimella et al. (70) found that among 2,377 older patients with nondiabetic CKD, lower urinary UMOD levels were associated with increased risk of mortality and cardiovascular events after a median follow-up time of 3.8 years. Further studies in patients with cardiovascular diseases demonstrated that plasma UMOD was associated with cardiovascular mortality independent of baseline renal function, thus making it a promising cardiovascular and renal biomarker (150).

#### 3.2.3 Markers of inflammation and fibrosis

Activation of inflammatory pathways in the kidney and recruitment of inflammatory cells at the site of injury are initial reactions to kidney damage (151, 152). Increase in inflammatory markers are frequently reported in CKD and correlate with eGFR changes (151, 153, 154). Furthermore, inflammation increases morbidity and worsens outcomes in patients with CKD (152) and may lead to development of fibrosis (155). Summary of findings from longitudinal studies exploring the role of inflammatory markers in CKD among middle aged and older people is reported in Table 3 (81, 122, 151, 156–170).

Interleukin-6 (IL-6) and Tumor Necrosis Factor-α (TNF-α) are two important mediators of inflammation that are extensively studied as coordinators of the inflammatory responses in AKI and CKD (153, 171). Proteomic analyses have shown how levels of these cytokines start increasing since early CKD stages (172) and may significantly contribute to disease progression and development of complications and poor health outcomes (151, 157, 164, 167, 173). Recent evidence also suggests that IL-6 may increase the production of FGF-23 levels in both AKI and CKD (174). Baseline IL-6 and TNF-α levels have recently been associated with longitudinal risk of eGFR decline or CKD progression in 3430 patients with baseline eGFR reduction (151); studies investigating the relationship between biomarker levels and all-cause/CV mortality are more numerous; most of them showed that both serum IL-6 and TNF-α levels were able to predict all-cause and CV mortality in older adults with and without CKD (164, 167). However, in one study including patients with CKD and ESKD, IL-6 only showed a significant correlation with calcium score and improved prognostic risk stratification compared with traditional CV risk factors (164). Conversely, in the study by Sun et al. (157), while both cytokines were independent predictors of all-cause mortality among older patients with advanced CKD, only low TNF-α levels were associated with CV mortality.

Interleukin-8 (IL-8) and Interleukin-18 (IL-18) are another two potential mediators of inflammatory response in CKD (175) and may both contribute to renal function decline (122, 172, 176) in CKD; IL-18 is a proinflammatory cytokine belonging to the IL-1 superfamily, and mediates infiltration of neutrophils and monocytes into the renal parenchyma in response to acute tubular injury (177). Both cytokines have been positively associated with structural damage to podocytes, peritubular dysfunction, and albuminuria in patients with type 2 diabetes, and negatively associated with eGFR (176). However, only in one study including 2,428 older adults with CKD and a mean age of 73 years, urinary IL-18 baseline levels were associated with eGFR decline and incidence of ESKD (122).

Soluble receptors of TNF-α, namely TNFR1 and TNFR2, are markers of low-grade inflammation and have recently been associated with kidney disease (178). These soluble proteins are part of the TNF receptor superfamily and are released in the blood from their membrane-bound main receptors and play a significant role in the advancement of atherosclerosis and diabetic kidney diseases (159, 178–180). Regulation of inflammatory responses and apoptosis through activation of nuclear factor kappa B (NF-κB) is achieved by TNF-α binding to TNFRs. Previous research has demonstrated a strong correlation between elevated levels of circulating TNFRs and the progression of diabetic nephropathy to CKD stage 3 and ESKD, as well as overall mortality (158, 179, 181). Such evidence has been recently corroborated by results of a post-hoc analysis of the CANVAS study: in patients with type 2 diabetes and albuminuria, baseline TNFR1 and 2 levels predicted CKD progression over time (169).

Other factors involved in CKD-related inflammatory response included all the chemokines during the inflammatory process, in response to cytokines such as TNF-α and interleukin-1 beta (IL-1β). Among them, monocyte chemoattractant protein-1 (MCP-1) interacts with the chemokine receptor 2 (CCR2) and stimulate the attraction of monocytes and macrophages from the bloodstream and surrounding tissues (182). To date, serum and urinary MCP-1 concentrations resulted to significantly predict the incidence of microalbuminuria in patients with diabetes mellitus and normoalbuminuria (168); furthermore, urinary levels of the biomarker may be useful to predict disease progression and ESKD in patients with CKD (122).

Other two important factors involved in chronic inflammation in CKD are the growth differentiation factor-15 (GDF-15) and the pentraxin-3 (PTX 3). GDF-15 is a member of the TGF-β superfamily and is induced in response to tissue injury. Increase in GDF-15 levels was previously associated with eGFR decline and CKD progression to ESKD in the Framingham study (183) and in two independent cohorts of 521 older adults with CKD (160) and in 219 older adults with CKD from the GCKD study (184). These results were confirmed in a large cohort of over 3,000 patients from the CRIC study, where GDF-15 outweighs NT-proBNP capacity to predict CKD progression (185). PTX 3 is secreted by central and peripheral immune cells in response to injury and is emerging as an interesting biomarker of CKD incidence and progression. A cross-sectional study involving older Korean individuals found an association between PTX-3 levels and risk of CKD (179). This association was also confirmed in longitudinal analyses; indeed, in a prospective study conducted by Sjöberg et al. (156), serum PTX3 levels were associated with 5-year incidence of CKD in two distinct cohorts of community-dwelling older patients; furthermore, when measured in a small sample of 78 patients with stage 5 CKD, serum PTX3 levels predicted all-cause and CV mortality, and significantly improved the cardiovascular risk stratification compared with classical CV risk factors (161). Predictive accuracy of PTX3 was even higher than that of hsCRP, IL6, 18 and TNFR1. Similar findings were observed by Valente et al. (165).

Finally, the soluble urokinase plasminogen activator receptor (SuPAR) is a protein that enters the bloodstream during inflammation when the urokinase plasminogen activator receptor is cleaved on podocytes and endothelial cells (186). SuPAR was identified as a disease activity marker in focal segmental glomerulosclerosis, although it was later studied in patients with AKI, DKD and CKD (186).

Acute and chronic injuries to the kidneys can trigger reparative processes or activation of progressive inflammatory pathways, ultimately resulting in fibrosis (155). Pathways leading to renal fibrosis are tightly regulated by some urinary biomarkers. Among them, the chitinase-3-D like protein 1, also known as YKL-40, has increasingly gained attention in the last years. This inflammatory glycoprotein of 40-kDa is secreted by many inflammatory cells and may serve as a signaling molecule for detection of responses to cellular damage (166). Similarly, the monocyte chemotactic protein (MCP-1) may represent a bridge between chronic inflammation and renal fibrosis (187). Previous studies have shown YKL-40 and MCP-1 prognostic potential in CKD patients (Table 2). Indeed, among older Veterans with albuminuria and diabetes, both urinary biomarkers have been associated with eGFR decline and mortality (124); furthermore, in a sub-cohort of CKD patients from the SPRINT study, YKL-40 and MCP-1 levels predicted CKD progression and the onset of CKD over 3.8 years of median follow-up time (122). Similar results were obtained in a hospitalized cohort of 1,538 older patients, where high urinary MCP-1 and YKL-40 concentrations were associated with eGFR decline, CKD progression and all-cause mortality, over a median follow-up time of 4.3 years (125).

## 4 Interplay between functional impairment and CKD

**Figure 3:**
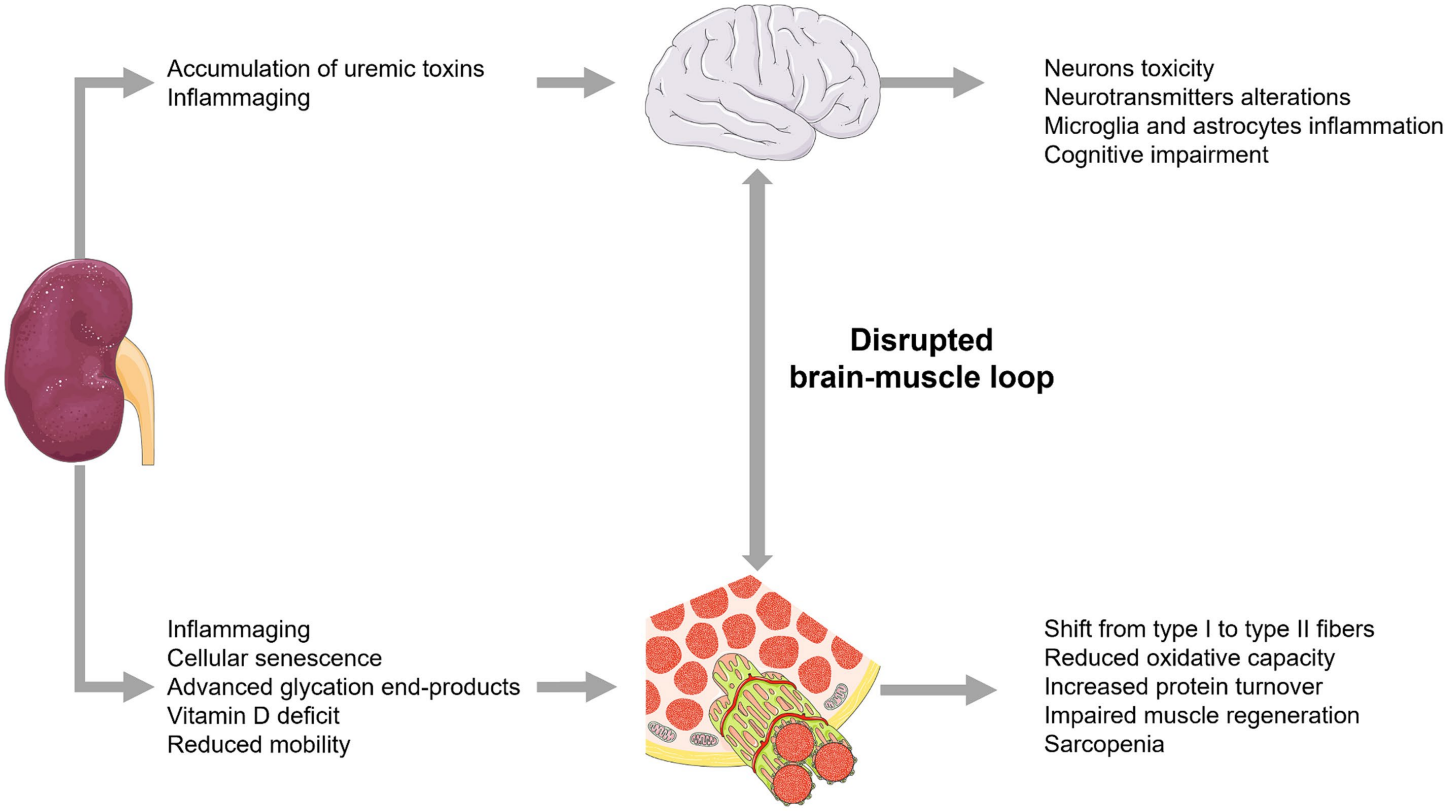
Pathogenic links between CKD, sarcopenia, physical and cognitive frailty.

Impairment of functional status in older individuals has a profound impact on the outcome of older individuals with CKD. Many geriatric syndromes such as sarcopenia, frailty and cognitive impairment may occur during CKD (Figure 3) and may impact biomarker’s levels and accuracy in diagnosing and monitoring CKD (188–195).

### 4.1 Biomarkers linking aging, sarcopenia, frailty, and CKD

Sarcopenia is common in older individuals and has an increased prevalence in patients with CKD, because impaired renal function disrupts muscle biogenesis (196). Both inflammaging and altered renal function upregulate proinflammatory pathways leading to increased production of IL-6, which is a marker of sarcopenia (197–199). Elevated levels of proinflammatory cytokines have been found to be increased in muscle biopsies from patients and in mouse models of CKD (200). Huang et al. (201) demonstrated that activation of IL-6/JAK2/STAT3 pathway increases denervated skeletal muscle atrophy. Its inhibition reduces skeletal muscle atrophy and may exert renoprotective effects by attenuating renal inflammation and renal injury (202, 203). However, such biomarker changes are nonspecific and may also be strongly influenced by age-related low-grade chronic inflammation process, known as inflammaging; cellular senescence also contributes to sarcopenia by regulating muscle dysfunction, primarily through stimulation of the p53/p21CIP1 and p16INK4a/pRB pathways, which are up-regulated during aging and contribute to irreversible growth arrest (204).

Other biomarkers bridging CKD, sarcopenia, and aging are represented by Klotho protein, AGEs, and vitamin D.

Klotho is an anti-ageing factor secreted by renal tubular cells and *in vivo* studies Klotho-deficient mice exhibit significant muscle loss (205); furthermore, age-related decrease in Klotho protein levels may contribute to the onset of CKD, representing a potentially early biomarker of the disease (97, 98). AGEs represent another relevant link between CKD and sarcopenia in older individuals; indeed, their ageing-induced accumulation in individuals with impaired renal function is exacerbated by inflammaging, characterized by an imbalance between oxidant and anti-oxidant mechanisms (206), which leads to further increases in reactive oxygen species production, inflammation and fibrosis (206), which can exacerbate muscle breakdown.

Another common factor of CKD and sarcopenia is represented by relative or absolute vitamin D deficiency. In particular, decreased activation of vitamin D by impaired kidneys leads to decreased pancreatic insulin secretion and blunted stimulation of protein synthesis (207); additionally, vitamin D deficiency can upregulate the ubiquitin-proteasome system (UPS) leading to protein catabolism and muscle breakdown (208).

Increased catabolism, insulin resistance, and vitamin D deficiency also contribute to linking CKD to the onset of frailty in older patients and its progression with the loss of renal function (209). In dialysis-dependent CKD, frailty is further exacerbated by metabolic acidosis, inflammation, and malnutrition. However, these mechanisms are complex and their role in development of frailty in CKD needs to be more clearly addressed; indeed, on one hand, the imbalance between decreased protective factors (i.e., antioxidant defenses, vascular capacity, Klotho and PPARγ) and increased stress factors (i.e., hypoxia, overexpression of proinflammatory cytokines) is recognized as a key element to development of sarcopenia, anemia, CKD, and frailty (5, 7); on the other hand, some evidence showed that the association between CKD and frailty was independent of C-reactive protein, low muscle mass and body mass index (210).

CKD patients are at increased risk of developing cognitive impairment, especially in advanced stages of the disease; indeed, the accumulation of uraemic toxins in the bloodstream due to the reduced ability of the kidneys to remove toxic compounds drives neurotoxicity and development of cognitive deterioration (211, 212). Indeed, uric acid, indoxyl sulphate, p-cresyl sulphate, interleukin-1β (IL-1β), IL-6 and tumour necrosis factor-α (TNF-α) are thought to play a more important role in the development of brain-kidney dysfunction (213). Several uremic toxins may affect dopaminergic neurons in the brain, likely contributing to the pathophysiology of CKD-related sleep abnormalities and restless syndrome (214). Additionally, increased intra-brain levels and low circulating levels of L-serine were recently observed in cognitively impaired CKD patients (215). Experimental evidence also suggests that cognitive symptoms induced by CKD-related uremic compounds accumulation may be at least partly caused by an increased glutamatergic transmission in the hippocampus (216). Furthermore, indoxyl sulphate was found to induce NLRP3 inflammasome-mediated microglia and astrocytic inflammation, which in turn may contribute to cognitive impairment (217). Finally, the above signalling pathways can also accelerate physical impairment by disrupting the functionality of the brain-muscle loop (218) and further contribute to sarcopenia and frailty.

### 4.2 Clinical evidence

The relationship between CKD and sarcopenia has been extensively investigated. Indeed, sarcopenia is highly prevalent in patients with advanced CKD and is closely linked to decline in GFR (219, 220). In a prospective observational study conducted in a population of 322 patients (123 hospitalized CKD patients and 57 healthy volunteers), it was found that patients with CKD were more prone to sarcopenia than healthy volunteers. Furthermore, Foley et al. (221) showed that prevalence of sarcopenia in US older adults increases from 26.6% when estimated glomerular filtration rate (eGFR) is ≥90 mL/min/1.73 m^2^, to 38.9% when eGFR is 60–89 to more than 60% for eGFR <60 mL/min. Such findings have been recently confirmed by Formiga et al. (222) in a large cohort of community-dwelling older adults from 7 European countries; along with evidence of a graded increase in prevalence of sarcopenia from CKD stages 1–2 to more advanced 3–4, authors showed that the interplay between eGFR and muscle loss was independent from equations used to estimate GFR (95, 222). Another cross-sectional study conducted in 95 dialysis patients revealed that sarcopenia is highly prevalent in older patients with ESKD, with a prevalence of 37.0% in men and 29.3% in women patients. In addition, this study measured various proinflammatory markers such as IL-6. The authors demonstrated that IL-6 was significantly associated with sarcopenia than those without (223), confirming that inflammaging may represent a valuable link between CKD and sarcopenia (201, 224).

Furthermore, a bidirectional interplay exists also between albuminuria and sarcopenia; indeed, albuminuria is considered a risk factor for sarcopenia, even in the absence of GFR decline, and independent of diabetes and hypertension (222, 225). This finding was retrospectively showed also in older patients with diabetes, where sarcopenia was associated with increased risk of albuminuria progression (226); shared underlying mechanisms (i.e., inflammaging, insulin resistance and renin angiotensin system activation) may explain the associations between sarcopenia, GFR, and ACR (227, 228).

Co-occurrence of CKD and sarcopenia has many prognostic implications; indeed, pooled analysis from a recent metanalysis, showed that both low muscle mass, strength and physical performance was associated with increased risk of mortality in CKD older adults (229). Another meta-analysis in patients with ESKD revealed that presence of sarcopenia significantly increases the risk of overall mortality and cardiovascular events (230).

As well as with sarcopenia, also the relationship between frailty and CKD has been widely studied in recent years. In a recent systematic review and meta-analysis, an update of seven previous meta-analyses published between 2017 and 2021 was conducted to address certain shortcomings or limitations. Among the 139 articles that met the eligibility criteria for the meta-analysis, and included a total of 1,675,482 participants, the results showed that 34.5% of CKD patients had signs of frailty, and 39.4% had prefrailty symptoms. Compared to non-frail patients, frail individuals had a 94.1% increased risk of death, while prefrail patients had a 34.5% increased risk of death (209). According to this study, about one-third of patients with CKD were affected by frailty. Furthermore, a recent meta-analysis of 7 studies showed that several eGFR equations predicted functional disability with the same strength, but Cystatin C-based equations improved prediction of incident disability in one study (231). Similarly, Mielke et al. (210) provided convincing evidence that combined use of cystatin C-based eGFR and albuminuria was associated with frailty progression in community-dwelling older adults (232). Therefore, frailty in CKD needs to be taken into account to reduce negative clinical outcomes and provide appropriate guidelines for this population (233).

Prevalence of physical frailty in CKD is however highly variable depending on age and setting of older population studied (234) as well as on the tool used to diagnose frailty (235); indeed, it reaches 11% in the general community dwelling older population, while rates are much higher in dialysis-dependent CKD patients ranging from 46% to over 60%, respectively (235). The prevalence of frailty, as measured by the physical frailty phenotype (PFP) is around 14% in CKD stages 1–3, and patients with CKD stage 3b or higher are almost six times more likely to be classified as frail (209); in dialysis patients, prevalence ranges from 30 to 82% depending on study setting and assessment tool (234). In any case, physical frailty negatively affects clinical outcomes of older patients with CKD of any stage (236) and was found to be a stronger predictor of adverse clinical outcomes than estimated glomerular filtration rate (237). Frailty and renal function are independently associated with symptom burden in CKD patients and contribute to potentially poorer quality of life.

A systematic review (238) confirms the correlation between frailty, CKD, and adverse clinical outcomes. This review identified 7 studies, including a total of 20,332 patients which demonstrated that CKD patients had a two-fold higher risk of physical frailty compared to healthy patients. Indeed, two of these studies assessed that frailty in CKD patients was associated with a significant risk of mortality. Another meta-analysis (239) of 18 longitudinal studies involving 24,788 patients assessed frailty as a negative predictor of adverse clinical outcomes in CKD patients. The prevalence of frailty in patients with CKD was 41.8%. Specifically, this study revealed that frailty is an independent predictor of all-cause mortality, all-cause hospitalization, and falls in patients with CKD (209). Furthermore, a study conducted on 1,830 older individuals, showed that eGFR decrease was associated with increased 2-year risk of frailty, independent of biomarkers used to assess kidney function (e.g., creatinine, cystatin C, beta-2 microglobulin) (240).

People with CKD may have a higher risk of cognitive frailty than people without the disease. This was investigated in a meta-analysis that included 54,779 participants from cross-sectional and longitudinal studies. It was the first study to show an association between CKD and cognitive impairments. However, it is important to note that while this meta-analysis suggest an association, there may still be contradictions on this topic (241), mainly due to the setting of populations studied and the screening method for cognitive status. A recent cross-sectional analysis of community-dwelling older adults showed that cognitive status, as measured through the Mini-Mental State Examination (MMSE) did not differ across CKD stages (242); conversely, a cross-sectional study in hospitalized older patients with CKD has suggested that the prevalence of cognitive frailty was relatively high in older patients with CKD (15.2%) and may increase with CKD progression (243). In line with previous findings, a cross-sectional study conducted in China showed that cognitive frailty manifested with a prevalence of 21.9% in older CKD patients (244). Furthermore, recently Scheppach et al. (245) found that eGFR and uACR were associated with increased risk of developing structural brain abnormalities visible on magnetic resonance imaging (MRI), mainly consisting in brain volume reduction, microhemorrhages and infarcts; interestingly, CKD-associated brain atrophy was not selective for regions usually involved in Alzheimer’s disease (245).

However, the relatively high burden of sarcopenia and frailty in CKD patients is exacerbated by their influence on biomarker levels (e.g., falsely low creatinine due to sarcopenia), that lead to potentially inaccurate CKD diagnosis in patients with geriatrics syndromes. This kind of patients likely requires other measures of kidney function not biased by reduced muscle mass and/or cognitive and physical frailty.

## 5 Future perspectives

The global burden of CKD among older individuals is dramatically increasing worldwide, mainly due to the escalating prevalence of hypertension and diabetes mellitus and the increased life expectancy in patients with cardiovascular diseases (246, 247).

Adequate GFR estimation in older individuals and age-adaptation of CKD is currently a topic of discussion among experts (248); recognition of eGFR <45 mL/min/1.73 m^2^ as a critical threshold to define older patients with CKD stage 3a would certainly prevent overdiagnosis and create diagnostic and classification systems tailored to older patients (249). However, as proposed by Levey et al. (250), age-calibration of CKD definition would be more feasible to capture the overall burden and complexity of CKD in geriatric populations. Impaired physical and cognitive performance, physical and cognitive frailty, sarcopenia, and malnutrition are all known to influence kidney function and may impact prognosis in the older population. A recent individual participant-data meta-analysis including 114 cohorts of over 27 million individuals showed that eGFR 45–59 mL/min based on serum creatinine levels has been associated with increased hospitalization risk compared to higher eGFR. The authors used two primary formulas to estimate GFR: creatinine-based eGFR and creatinine and cystatin C-based eGFR (eGFRcr-cys) (251). Implementation of comprehensive geriatric assessment in everyday clinical practice could help detect patients at risk of poor outcomes as a result of complex interplay between CKD, functional status (both physical and cognitive), sarcopenia, and malnutrition.

In order to improve timing for an early CKD detection, several biomarkers are currently under study. Despite the relative abundance and diversity of biomarkers potentially useful for the diagnosis and prognostic stratification of older individuals with CKD, there is still lack of accurate biomarkers among older individuals; this may be attributed to several reasons; first, most studies included cohorts of wide age ranges and with specific diseases (e.g., diabetes mellitus and heart failure) and only a minority specifically targeted older populations (Table 4); second, even when considering studies conducted in geriatric populations, most biomarkers did not significantly or importantly improve accuracy in predicting poor outcomes in such populations; finally, chronic diseases such as cardiovascular disease, diabetes, and autoimmune disorders often induce systemic inflammation, which can exacerbate renal dysfunction. These systemic inflammatory responses can confound the interpretation of renal biomarkers, as elevated levels may reflect not only intrinsic kidney disease but also the inflammatory burden from comorbid conditions. Consequently, it becomes challenging to distinguish between primary renal pathology and secondary effects of systemic inflammation, necessitating a comprehensive approach to evaluating renal biomarkers in elderly patients with multiple chronic illnesses.

**Table 4:** Summary of evidence from studies assessing biomarkers of kidney function in populations aged 60 years or older.

Biomarkers	Type	Outcomes in geriatric populations	Results
Plasma Klotho	Glomerular	eGFR decline (Drew et al.)	Lower levels were associated with eGFR decline
Urinary B2M	Glomerular	All-cause and CV mortality, MACE (Garimella et al.)	Positively associated with all outcomes
Plasma NGAL	Tubular	Acute and 5-year eGFR decline, risk of hospitalization and mortality (Lim et al. 2015; Hasegawa et al. 2016)	Positively associated with study outcomes
Urinary NGAL	Tubular	CKD progression (Seibert et al. 2018)	Not associated
Urinary KIM-1	Tubular	CKD progression (Seibert et al. 2018), eGFR decline and progression to ESKD (Malhotra et al. 2020), all-cause and disease-specific mortality (Vasquez-Rios et al. 2023)	Positively associated with eGFR decline, progression to ESKD, and increased risk of mortality
Serum UMOD	Tubular	ESKD (Steubl et al. 2019)	Positively associated with the outcome
Urinary UMOD	Tubular	All-cause and CV mortality, MACE (Garimella et al. 2019; Vasquez-Rios G et al. 2023)	Positively associated all outcomes
Urinary α_1_ microglobulin	Tubular	All-cause and CV mortality, MACE (Garimella et al. 2019)	Positively associated with all outcomes
Urinary MCP-1	Tubular	eGFR decline, progression to ESKD (Malhotra et al. 2020), all-cause and disease-specific mortality (Vasquez-Rios et al. 2023)	Positively associated with eGFR decline, progression to ESKD, and cardiovascular mortality
Urinary YKL-40	Tubular	eGFR decline, progression to ESKD (Malhotra et al. 2020), all-cause and disease-specific mortality (Vasquez-Rios et al. 2023)	Positively associated with eGFR decline, progression to ESKD, and increased risk of all-cause mortality
Urinary IL-18	Inflammatory	eGFR decline and progression to ESKD (Malhotra et al. 2020)	Positively associated with study outcomes
Serum PTX3		Incident CKD (Sjöberg et al. 2016)	Positively associated with the outcome
Serum TNFR1	Inflammatory	CKD progression in diabetic nephropathy (Fernàndez-Juàrez et al. 2017)	Positively associated with the outcome

B2M, β2 microglobulin; CKD, chronic kidney disease; eGFR, estimated glomerular filtration rate; ESKD, end-stage kidney disease; KIM-1, kidney injury molecule-1; MCP-1, monocyte chemotactic protein-1; NGAL, neutrophil gelatinase-associated lipocalin; PTX3, pentraxin-related protein 3; TNFR1, tumor necrosis factor receptor 1; UMOD, uromodulin; YKL, chitinase-3-like protein 1.

For all these reasons, eGFR and ACR are still the best available cost-effective methods for CKD diagnosis and prognostic risk stratification in geriatric populations, given their higher cost-effectiveness and availability across clinical settings compared to novel biomarkers. Further studies specifically focused on geriatric populations with CKD are required to improve characterization and diagnosis of CKD in older individuals.

## Author contributions

LM: Writing – original draft, Writing – review & editing. MDD: Conceptualization, Writing – review & editing. EF: Writing – review & editing. GIG: Writing – review & editing. MV: Writing – review & editing. AB: Writing – review & editing. PF: Writing – review & editing. FL: Writing – review & editing. AC: Writing – original draft, Writing – review & editing. GG: Writing – review & editing. DS: Writing – review & editing. LS: Conceptualization, Supervision, Writing – original draft, Writing – review & editing.

## References

[1] Johansen KL Chertow GM Gilbertson DT Herzog CA Ishani A Israni AK. US renal data system 2021 annual data report: epidemiology of kidney disease in the United States. Am J Kidney Dis. (2022) 79:A8–A12. doi: 10.1053/j.ajkd.2022.02.001, PMID: 35331382 PMC8935019

[2] Fang Y Gong AY Haller ST Dworkin LD Liu Z Gong R. The ageing kidney: molecular mechanisms and clinical implications. Ageing Res Rev. (2020) 63:101151. doi: 10.1016/j.arr.2020.101151, PMID: 32835891 PMC7595250

[3] Ray N Reddy PH. Structural and physiological changes of the kidney with age and its impact on chronic conditions and COVID-19. Ageing Res Rev. (2023) 88:101932. doi: 10.1016/j.arr.2023.101932, PMID: 37031725 PMC10081878

[4] Soraci L Cherubini A Paoletti L Filippelli G Luciani F Laganà P. Safety and tolerability of antimicrobial agents in the older patient. Drugs Aging. (2023) 40:499–526. doi: 10.1007/s40266-023-01019-3, PMID: 36976501 PMC10043546

[5] Ortiz A Mattace-Raso F Soler MJ Fouque D. Ageing meets kidney disease. Nephrol Dial Transplant. (2023) 38:523–6. doi: 10.1093/ndt/gfac199, PMID: 35768068 PMC9976735

[6] Alfano G Perrone R Fontana F Ligabue G Giovanella S Ferrari A. Rethinking chronic kidney disease in the aging population. Life. (2022) 12:1724. doi: 10.3390/life12111724, PMID: 36362879 PMC9699322

[7] Aucella F Corsonello A Leosco D Brunori G Gesualdo L Antonelli-Incalzi R. Beyond chronic kidney disease: the diagnosis of renal disease in the elderly as an unmet need. A position paper endorsed by Italian Society of Nephrology (SIN) and Italian Society of Geriatrics and Gerontology (SIGG). J Nephrol. (2019) 32:165–76. doi: 10.1007/s40620-019-00584-4, PMID: 30659521 PMC6423311

[8] Webster AC Nagler EV Morton RL Masson P. Chronic kidney disease. Lancet. (2017) 389:1238–52. doi: 10.1016/S0140-6736(16)32064-5 27887750

[9] Glassock RJ Rule AD. The implications of anatomical and functional changes of the aging kidney: with an emphasis on the glomeruli. Kidney Int. (2012) 82:270–7. doi: 10.1038/ki.2012.65, PMID: 22437416 PMC3513938

[10] Lindeman RD Tobin J Shock NW. Longitudinal studies on the rate of decline in renal function with age. J Am Geriatr Soc. (1985) 33:278–85. doi: 10.1111/j.1532-5415.1985.tb07117.x 3989190

[11] Wetzels JF Kiemeney LALM Swinkels DW Willems HL Heijer M. Age- and gender-specific reference values of estimated GFR in Caucasians: the Nijmegen biomedical study. Kidney Int. (2007) 72:632–7. doi: 10.1038/sj.ki.5002374, PMID: 17568781

[12] Glassock RJ Denic A Rule AD. The conundrums of chronic kidney disease and aging. J Nephrol. (2017) 30:477–83. doi: 10.1007/s40620-016-0362-x, PMID: 27885585

[13] Denic A Mathew J Lerman LO Lieske JC Larson JJ Alexander MP. Single-nephron glomerular filtration rate in healthy adults. N Engl J Med. (2017) 376:2349–57. doi: 10.1056/NEJMoa1614329, PMID: 28614683 PMC5664219

[14] Esposito C Dal Canton A. Functional changes in the aging kidney. J Nephrol. (2010) 23:S41–5. 20872370

[15] Schuck O Nadvornikova H. Short acidification test and its interpretation with respect to age. Nephron. (1987) 46:215–6. doi: 10.1159/000184348, PMID: 3600935

[16] Corsonello A Freiberger E Lattanzio F. The screening for chronic kidney disease among older people across Europe (SCOPE) project: findings from cross-sectional analysis. BMC Geriatr. (2020) 20:316. doi: 10.1186/s12877-020-01701-w, PMID: 33008358 PMC7531078

[17] Merchant RA Vathsala A. Healthy aging and chronic kidney disease. Kidney Res Clin Pract. (2022) 41:644–56. doi: 10.23876/j.krcp.22.112, PMID: 36328991 PMC9731776

[18] Rule AD Glassock RJ. GFR estimating equations: getting closer to the truth? Clin J Am Soc Nephrol. (2013) 8:1414–20. doi: 10.2215/cjn.01240213, PMID: 23704300 PMC3731897

[19] Patel SS Molnar MZ Tayek JA Ix JH Noori N Benner D. Serum creatinine as a marker of muscle mass in chronic kidney disease: results of a cross-sectional study and review of literature. J Cachexia Sarcopenia Muscle. (2013) 4:19–29. doi: 10.1007/s13539-012-0079-1, PMID: 22777757 PMC3581614

[20] Branten AJ Vervoort G Wetzels JF. Serum creatinine is a poor marker of GFR in nephrotic syndrome. Nephrol Dial Transplant. (2005) 20:707–11. doi: 10.1093/ndt/gfh719, PMID: 15713698

[21] Goek O-N Döring A Gieger C Heier M Koenig W Prehn C. Serum metabolite concentrations and decreased GFR in the general population. Am J Kidney Dis. (2012) 60:197–206. doi: 10.1053/j.ajkd.2012.01.014, PMID: 22464876

[22] Levey AS Stevens LA Schmid CH Zhang YL Castro AF 3rd Feldman HI. A new equation to estimate glomerular filtration rate. Ann Intern Med. (2009) 150:604–12. doi: 10.7326/0003-4819-150-9-200905050-00006, PMID: 19414839 PMC2763564

[23] Inker LA Eneanya ND Coresh J Tighiouart H Wang D Sang Y. New creatinine- and cystatin C-based equations to estimate GFR without race. N Engl J Med. (2021) 385:1737–49. doi: 10.1056/NEJMoa2102953, PMID: 34554658 PMC8822996

[24] Montesanto A de Rango F Berardelli M Mari V Lattanzio F Passarino G. Glomerular filtration rate in the elderly and in the oldest old: correlation with frailty and mortality. Age. (2014) 36:9641. doi: 10.1007/s11357-014-9641-4, PMID: 24664801 PMC4082598

[25] Cox HJ Bhandari S Rigby AS Kilpatrick ES. Mortality at low and high estimated glomerular filtration rate values: a ‘U’ shaped curve. Nephron Clin Pract. (2008) 110:c67–72. doi: 10.1159/000151720, PMID: 18758185

[26] Peters R Beckett N Poulter R Burch L Narkiewicz K Fagard R. Kidney function in the very elderly with hypertension: data from the hypertension in the very elderly (HYVET) trial. Age Ageing. (2013) 42:253–8. doi: 10.1093/ageing/afs109, PMID: 22910302

[27] Schaeffner ES Ebert N Delanaye P Frei U Gaedeke J Jakob O. Two novel equations to estimate kidney function in persons aged 70 years or older. Ann Intern Med. (2012) 157:471–81. doi: 10.7326/0003-4819-157-7-201210020-00003 23027318

[28] Pottel H Hoste L Dubourg L Ebert N Schaeffner E Eriksen BO. An estimated glomerular filtration rate equation for the full age spectrum. Nephrol Dial Transplant. (2016) 31:798–806. doi: 10.1093/ndt/gfv454, PMID: 26932693 PMC4848755

[29] Oscanoa TJ Amado JP Romero-Ortuno R Hidalgo JA. Estimation of the glomerular filtration rate in older individuals with serum creatinine-based equations: a systematic comparison between CKD-EPI and BIS1. Arch Gerontol Geriatr. (2018) 75:139–45. doi: 10.1016/j.archger.2017.12.007 29304508

[30] Corsonello A Roller-Wirnsberger R Wirnsberger G Ärnlöv J Carlsson AC Tap L. Clinical implications of estimating glomerular filtration rate with three different equations among older people. Preliminary results of the project “screening for chronic kidney disease among older people across Europe (SCOPE)”. J Clin Med. (2020) 9:294. doi: 10.3390/jcm9020294, PMID: 31973029 PMC7074235

[31] Ma Y Shen X Yong Z Wei L Zhao W. Comparison of glomerular filtration rate estimating equations in older adults: a systematic review and meta-analysis. Arch Gerontol Geriatr. (2023) 114:105107. doi: 10.1016/j.archger.2023.105107, PMID: 37379796

[32] Raman M Middleton RJ Kalra PA Green D. Estimating renal function in old people: an in-depth review. Int Urol Nephrol. (2017) 49:1979–88. doi: 10.1007/s11255-017-1682-z, PMID: 28913589 PMC5643354

[33] da Silva Selistre L Rech DL de Souza V Iwaz J Lemoine S Dubourg L. Diagnostic performance of creatinine-based equations for estimating glomerular filtration rate in adults 65 years and older. JAMA Intern Med. (2019) 179:796–804. doi: 10.1001/jamainternmed.2019.0223, PMID: 31034005 PMC6547158

[34] Fehrman-Ekholm I Seeberger A Björk J Sterner G. Serum cystatin C: a useful marker of kidney function in very old people. Scand J Clin Lab Invest. (2009) 69:606–11. doi: 10.1080/00365510903015771, PMID: 19517296

[35] Ren C Su H Tao J Xie Y Zhang X Guo Q. Sarcopenia index based on serum creatinine and cystatin C is associated with mortality, nutritional risk/malnutrition and sarcopenia in older patients. Clin Interv Aging. (2022) 17:211–21. doi: 10.2147/cia.S351068, PMID: 35256845 PMC8898017

[36] Wu Y Wang H Tong Y Zhang X Long Y Li Q. Sarcopenia index based on serum creatinine and cystatin C is associated with mortality in middle-aged and older adults in Chinese: a retrospective cohort study from the China health and retirement longitudinal study. Front Public Health. (2023) 11:1122922. doi: 10.3389/fpubh.2023.1122922, PMID: 37026117 PMC10071508

[37] Tang T Zhuo Y Xie L Wang H Yang M. Sarcopenia index based on serum creatinine and cystatin C is associated with 3-year mortality in hospitalized older patients. Sci Rep. (2020) 10:1260. doi: 10.1038/s41598-020-58304-z, PMID: 31988356 PMC6985114

[38] Inker LA Okparavero A. Cystatin C as a marker of glomerular filtration rate: prospects and limitations. Curr Opin Nephrol Hypertens. (2011) 20:631–9. doi: 10.1097/MNH.0b013e32834b8850 21926620

[39] Gharaibeh KA Hamadah AM El-Zoghby ZM Lieske JC Larson TS Leung N. Cystatin C predicts renal recovery earlier than creatinine among patients with acute kidney injury. Kidney Int Rep. (2018) 3:337–42. doi: 10.1016/j.ekir.2017.10.012, PMID: 29725637 PMC5932123

[40] Guan C Liang M Liu R Qin S He F Li J. Assessment of creatinine and cystatin C-based eGFR equations in Chinese older adults with chronic kidney disease. Int Urol Nephrol. (2018) 50:2229–38. doi: 10.1007/s11255-018-1909-7, PMID: 29948865

[41] Potok OA Rifkin DE Ix JH Shlipak MG Satish A Schneider A. Estimated GFR accuracy when cystatin C- and creatinine-based estimates are discrepant in older adults. Kidney Med. (2023) 5:100628. doi: 10.1016/j.xkme.2023.100628, PMID: 37168389 PMC10165149

[42] Iversen E Bodilsen AC Klausen HH Treldal C Andersen O Houlind MB. Kidney function estimates using cystatin C versus creatinine: impact on medication prescribing in acutely hospitalized elderly patients. Basic Clin Pharmacol Toxicol. (2019) 124:466–78. doi: 10.1111/bcpt.13156, PMID: 30372593

[43] Tavares J Santos J Silva F Oliveira J Malheiro J Campos A. Association between severe chronic kidney disease defined by cystatin-c and creatinine and clinical outcomes in an elderly population—an observational study. J Bras Nefrol. (2021) 43:165–72. doi: 10.1590/2175-8239-jbn-2020-0092 33258463 PMC8257284

[44] Cheang I Liao S Yao W Lu X Gao R Zhou Y. Cystatin C-based CKD-EPI estimated glomerular filtration rate equations as a better strategy for mortality stratification in acute heart failure: a STROBE-compliant prospective observational study. Medicine. (2020) 99:e22996. doi: 10.1097/md.0000000000022996, PMID: 33126378 PMC7598854

[45] Willey JZ Moon YP Husain SA Elkind MSV Sacco RL Wolf M. Creatinine versus cystatin C for renal function-based mortality prediction in an elderly cohort: the northern Manhattan study. PLoS One. (2020) 15:e0226509. doi: 10.1371/journal.pone.0226509, PMID: 31940363 PMC6961921

[46] Helmersson-Karlqvist J Lipcsey M Ärnlöv J Bell M Ravn B Dardashti A. Cystatin C predicts long term mortality better than creatinine in a nationwide study of intensive care patients. Sci Rep. (2021) 11:5882. doi: 10.1038/s41598-021-85370-8, PMID: 33723337 PMC7961058

[47] NICE. (2021). Chronic kidney disease: assessment and management. Available at: https://www.nice.org.uk/guidance/ng203 (Accessed December 20, 2023).

[48] Kusunoki H Tsuji S Kusukawa T Wada Y Tamaki K Nagai K. Relationships between cystatin C- and creatinine-based eGFR in Japanese rural community- dwelling older adults with sarcopenia. Clin Exp Nephrol. (2021) 25:231–9. doi: 10.1007/s10157-020-01981-x, PMID: 33090338 PMC7925493

[49] Carrero JJ Grams ME Sang Y Ärnlöv J Gasparini A Matsushita K. Albuminuria changes are associated with subsequent risk of end-stage renal disease and mortality. Kidney Int. (2017) 91:244–51. doi: 10.1016/j.kint.2016.09.037, PMID: 27927597 PMC5523054

[50] Levey AS Becker C Inker LA. Glomerular filtration rate and albuminuria for detection and staging of acute and chronic kidney disease in adults: a systematic review. JAMA. (2015) 313:837–46. doi: 10.1001/jama.2015.0602, PMID: 25710660 PMC4410363

[51] Lamb EJ MacKenzie F Stevens PE. How should proteinuria be detected and measured? Ann Clin Biochem. (2009) 46:205–17. doi: 10.1258/acb.2009.009007 19389884

[52] Stevens PE Levin A. Evaluation and management of chronic kidney disease: synopsis of the kidney disease: improving global outcomes 2012 clinical practice guideline. Ann Intern Med. (2013) 158:825–30. doi: 10.7326/0003-4819-158-11-201306040-00007, PMID: 23732715

[53] Martinez YV Benett I Lewington AJP Wierzbicki AS Guideline Committee. Chronic kidney disease: summary of updated NICE guidance. BMJ. (2021) 374:n1992. doi: 10.1136/bmj.n1992, PMID: 34489303

[54] Corsonello A Soraci L Ärnlöv J Carlsson AC Roller-Wirnsberger R Wirnsberger G. The relevance of geriatric assessments on the association between chronic kidney disease stages and mortality among older people: a secondary analysis of a multicentre cohort study. Age Ageing. (2022) 51:afac168. doi: 10.1093/ageing/afac168, PMID: 35871417 PMC9308988

[55] Melsom T Solbu MD Schei J Stefansson VTN Norvik JV Jenssen TG. Mild albuminuria is a risk factor for faster GFR decline in the nondiabetic population. Kidney Int Rep. (2018) 3:817–24. doi: 10.1016/j.ekir.2018.01.015, PMID: 29989017 PMC6035129

[56] Okubo A Nakashima A Doi S Doi T Ueno T Maeda K. High-normal albuminuria is strongly associated with incident chronic kidney disease in a nondiabetic population with normal range of albuminuria and normal kidney function. Clin Exp Nephrol. (2020) 24:435–43. doi: 10.1007/s10157-019-01842-2, PMID: 32076888

[57] Gerstein HC Mann JF Yi Q Zinman B Dinneen SF Hoogwerf B. Albuminuria and risk of cardiovascular events, death, and heart failure in diabetic and nondiabetic individuals. JAMA. (2001) 286:421–6. doi: 10.1001/jama.286.4.421, PMID: 11466120

[58] Ren F Li M Xu H Qin X Teng Y. Urine albumin-to-creatinine ratio within the normal range and risk of hypertension in the general population: a meta-analysis. J Clin Hypertens. (2021) 23:1284–90. doi: 10.1111/jch.14263, PMID: 34089300 PMC8678728

[59] Fangel MV Nielsen PB Kristensen JK Larsen TB Overvad TF Lip GYH. Albuminuria and risk of cardiovascular events and mortality in a general population of patients with type 2 diabetes without cardiovascular disease: a Danish cohort study. Am J Med. (2020) 133:e269–79. doi: 10.1016/j.amjmed.2019.10.042, PMID: 32205071

[60] Freedman BI Langefeld CD Lohman KK Bowden DW Carr JJ Rich SS. Relationship between albuminuria and cardiovascular disease in type 2 diabetes. J Am Soc Nephrol. (2005) 16:2156–61. doi: 10.1681/asn.2004100884 15872076

[61] Luis-Lima S Porrini E. An overview of errors and flaws of estimated GFR versus true GFR in patients with diabetes mellitus. Nephron. (2017) 136:287–91. doi: 10.1159/000453531, PMID: 27978513

[62] Wouters OJ O’Donoghue DJ Ritchie J Kanavos PG Narva AS. Early chronic kidney disease: diagnosis, management and models of care. Nat Rev Nephrol. (2015) 11:491–502. doi: 10.1038/nrneph.2015.85, PMID: 26055354 PMC4531835

[63] Rysz J Gluba-Brzózka A Franczyk B Jabłonowski Z Ciałkowska-Rysz A. Novel biomarkers in the diagnosis of chronic kidney disease and the prediction of its outcome. Int J Mol Sci. (2017) 18:1702. doi: 10.3390/ijms18081702, PMID: 28777303 PMC5578092

[64] Foster MC Coresh J Hsu CY Xie D Levey AS Nelson RG. Serum β-trace protein and β2-microglobulin as predictors of ESRD, mortality, and cardiovascular disease in adults with CKD in the chronic renal insufficiency cohort (CRIC) study. Am J Kidney Dis. (2016) 68:68–76. doi: 10.1053/j.ajkd.2016.01.015, PMID: 26948990 PMC4921300

[65] Drew DA Katz R Kritchevsky S Ix J Shlipak M Gutiérrez OM. Association between soluble klotho and change in kidney function: the health aging and body composition study. J Am Soc Nephrol. (2017) 28:1859–66. doi: 10.1681/asn.2016080828, PMID: 28104822 PMC5461794

[66] Rebholz CM Inker LA Chen Y Liang M Foster MC Eckfeldt JH. Risk of ESRD and mortality associated with change in filtration markers. Am J Kidney Dis. (2017) 70:551–60. doi: 10.1053/j.ajkd.2017.04.025, PMID: 28648303 PMC5610931

[67] Inker LA Coresh J Sang Y Hsu CY Foster MC Eckfeldt JH. Filtration markers as predictors of ESRD and mortality: individual participant data meta-analysis. Clin J Am Soc Nephrol. (2017) 12:69–78. doi: 10.2215/cjn.03660316, PMID: 28062677 PMC5220652

[68] Liu QF Yu LX Feng JH Sun Q Li SS Ye JM. The prognostic role of klotho in patients with chronic kidney disease: a systematic review and meta-analysis. Dis Markers. (2019) 2019:6468729–12. doi: 10.1155/2019/6468729, PMID: 31275449 PMC6589248

[69] Qian J Zhong J Yan M Cheng P Shi H Hao C. Circulating α-klotho is related to plasma aldosterone and its follow-up change predicts CKD progression. Kidney Blood Press Res. (2018) 43:836–46. doi: 10.1159/000490138, PMID: 29843135

[70] Garimella PS Lee AK Ambrosius WT Bhatt U Cheung AK Chonchol M. Markers of kidney tubule function and risk of cardiovascular disease events and mortality in the SPRINT trial. Eur Heart J. (2019) 40:3486–93. doi: 10.1093/eurheartj/ehz392, PMID: 31257404 PMC6837159

[71] Memmos E Sarafidis P Pateinakis P Tsiantoulas A Faitatzidou D Giamalis P. Soluble klotho is associated with mortality and cardiovascular events in hemodialysis. BMC Nephrol. (2019) 20:217. doi: 10.1186/s12882-019-1391-1, PMID: 31185930 PMC6560885

[72] Silva AP Mendes F Carias E Gonçalves RB Fragoso A Dias C. Plasmatic Klotho and FGF23 levels as biomarkers of CKD-associated cardiac disease in type 2 diabetic patients. Int J Mol Sci. (2019) 20:1536. doi: 10.3390/ijms20071536, PMID: 30934737 PMC6480092

[73] Leyssens K van Regenmortel N Roelant E Guerti K Couttenye MM Jorens PG. Beta-trace protein as a potential marker of acute kidney injury: a pilot study. Kidney Blood Press Res. (2021) 46:185–95. doi: 10.1159/000514173, PMID: 33784671

[74] Buyadaa O Salim A Morton JI Jandeleit-Dahm K Magliano DJ Shaw JE. Examining the factors contributing to the association between non-albuminuric CKD and a low rate of kidney function decline in diabetes. Ther Adv Endocrinol Metab. (2022) 13:20420188221083518. doi: 10.1177/20420188221083518, PMID: 35355954 PMC8958525

[75] Rebholz CM Grams ME Matsushita K Selvin E Coresh J. Change in novel filtration markers and risk of ESRD. Am J Kidney Dis. (2015) 66:47–54. doi: 10.1053/j.ajkd.2014.11.009, PMID: 25542414 PMC4478244

[76] Foster MC Levey AS Inker LA Shafi T Fan L Gudnason V. Non-GFR determinants of low-molecular-weight serum protein filtration markers in the elderly: AGES-kidney and MESA-kidney. Am J Kidney Dis. (2017) 70:406–14. doi: 10.1053/j.ajkd.2017.03.021, PMID: 28549536 PMC5572311

[77] Inker LA Tighiouart H Coresh J Foster MC Anderson AH Beck GJ. GFR estimation using β-trace protein and β2-microglobulin in CKD. Am J Kidney Dis. (2016) 67:40–8. doi: 10.1053/j.ajkd.2015.07.025, PMID: 26362696 PMC4695294

[78] George JA Gounden V. Novel glomerular filtration markers. Adv Clin Chem. (2019) 88:91–119. doi: 10.1016/bs.acc.2018.10.005 30612608

[79] Schwab S Kleine CE Bös D Bohmann S Strassburg CP Lutz P. Beta-trace protein as a potential biomarker of residual renal function in patients undergoing peritoneal dialysis. BMC Nephrol. (2021) 22:87. doi: 10.1186/s12882-021-02287-0, PMID: 33706697 PMC7953776

[80] Donadio C Bozzoli L. Urinary β-trace protein: a unique biomarker to screen early glomerular filtration rate impairment. Medicine. (2016) 95:e5553. doi: 10.1097/md.0000000000005553, PMID: 27930558 PMC5266030

[81] Bansal A Nigoskar S Thalquotra M. Comparison of BTP, NGAL, KIM-1, & ADMA biomarkers in CKD and non-CKD subjects. Int J Biochem Mol Biol. (2023) 14:32–9. PMID: 37456909 PMC10349297

[82] Chen N Shi H Zhang L Zuo L Xie J Xie D. GFR estimation using a panel of filtration markers in Shanghai and Beijing. Kidney Med. (2020) 2:172–80. doi: 10.1016/j.xkme.2019.11.004, PMID: 32734236 PMC7380432

[83] White CA Allen CM Akbari A Collier CP Holland DC Day AG. Comparison of the new and traditional CKD-EPI GFR estimation equations with urinary inulin clearance: a study of equation performance. Clin Chim Acta. (2019) 488:189–95. doi: 10.1016/j.cca.2018.11.019, PMID: 30445029

[84] Iversen E Bengaard AK Leegaard Andersen A Tavenier J Nielsen RL Juul-Larsen HG. Performance of panel-estimated GFR among hospitalized older adults. Am J Kidney Dis. (2023) 82:715–24. doi: 10.1053/j.ajkd.2023.05.004, PMID: 37516299

[85] Iversen E Boesby L Hansen D Houlind MB. Comparison of 24-hour urinary creatinine clearance and estimated glomerular filtration rate based on a panel of filtration markers in patients with chronic kidney disease. Pharmacol Res Perspect. (2022) 10:e01002. doi: 10.1002/prp2.1002, PMID: 36069238 PMC9449817

[86] Inker LA Couture SJ Tighiouart H Abraham AG Beck GJ Feldman HI. A new panel-estimated GFR, including β_2_-microglobulin and β-trace protein and not including race, developed in a diverse population. Am J Kidney Dis. (2021) 77:673–683.e1. doi: 10.1053/j.ajkd.2020.11.005, PMID: 33301877 PMC8102017

[87] Priem F Althaus H Birnbaum M Sinha P Conradt HS Jung K. Beta-trace protein in serum: a new marker of glomerular filtration rate in the creatinine-blind range. Clin Chem. (1999) 45:567–8. doi: 10.1093/clinchem/45.4.567, PMID: 10102918

[88] Motawi TK Shehata NI ElNokeety MM el-Emady YF. Potential serum biomarkers for early detection of diabetic nephropathy. Diabetes Res Clin Pract. (2018) 136:150–8. doi: 10.1016/j.diabres.2017.12.007 29253627

[89] Drüeke TB Massy ZA. Beta2-microglobulin. Semin Dial. (2009) 22:378–80. doi: 10.1111/j.1525-139X.2009.00584.x 19708985

[90] Stefanović V Djukanović L Čukuranović R Bukvić D Ležaić V Marić I. Beta2-microglobulin and alpha1-microglobulin as markers of Balkan endemic nephropathy, a worldwide disease. Ren Fail. (2011) 33:176–83. doi: 10.3109/0886022x.2011.552152, PMID: 21332340

[91] Foster MC Inker LA Hsu CY Eckfeldt JH Levey AS Pavkov ME. Filtration markers as predictors of ESRD and mortality in southwestern American Indians with type 2 diabetes. Am J Kidney Dis. (2015) 66:75–83. doi: 10.1053/j.ajkd.2015.01.013, PMID: 25773485 PMC4485524

[92] Shinkai S Chaves PH Fujiwara Y Watanabe S Shibata H Yoshida H. Beta2-microglobulin for risk stratification of total mortality in the elderly population: comparison with cystatin C and C-reactive protein. Arch Intern Med. (2008) 168:200–6. doi: 10.1001/archinternmed.2007.64, PMID: 18227369

[93] Gholaminejad A Moein S Roointan A Mortazavi M Nouri R Mansourian M. Circulating β2 and α1 microglobulins predict progression of nephropathy in diabetic patients: a meta-analysis of prospective cohort studies. Acta Diabetol. (2022) 59:1417–27. doi: 10.1007/s00592-022-01940-w, PMID: 35939238

[94] Pottel H Schaeffner E Ebert N. Evaluating the diagnostic value of rescaled β-trace protein in combination with serum creatinine and serum cystatin C in older adults. Clin Chim Acta. (2018) 480:206–13. doi: 10.1016/j.cca.2018.02.026, PMID: 29476732

[95] Moreno-González R Cruzado JM Corsonello A Fabbietti P Tap L Mattace-Raso F. Kidney function and other associated factors of sarcopenia in community-dwelling older adults: the SCOPE study. Eur J Intern Med. (2023) 123:81–93. doi: 10.1016/j.ejim.2023.12.002, PMID: 38103954

[96] Buchanan S Combet E Stenvinkel P Shiels PG. Klotho, aging, and the failing kidney. Front Endocrinol. (2020) 11:560. doi: 10.3389/fendo.2020.00560, PMID: 32982966 PMC7481361

[97] Zou D Wu W He Y Ma S Gao J. The role of klotho in chronic kidney disease. BMC Nephrol. (2018) 19:285. doi: 10.1186/s12882-018-1094-z, PMID: 30348110 PMC6198535

[98] Hu MC Kuro-o M Moe OW. Klotho and kidney disease. J Nephrol. (2010) 23:S136–44. 21170871 PMC3227531

[99] Wang Q Su W Shen Z Wang R. Correlation between soluble α-klotho and renal function in patients with chronic kidney disease: a review and meta-analysis. Biomed Res Int. (2018) 2018:9481475. doi: 10.1155/2018/9481475, PMID: 30159331 PMC6109492

[100] Kim HR Nam BY Kim DW Kang MW Han JH Lee MJ. Circulating α-klotho levels in CKD and relationship to progression. Am J Kidney Dis. (2013) 61:899–909. doi: 10.1053/j.ajkd.2013.01.024, PMID: 23540260

[101] Komaba H Lanske B. Role of klotho in bone and implication for CKD. Curr Opin Nephrol Hypertens. (2018) 27:298–304. doi: 10.1097/mnh.0000000000000423, PMID: 29697410

[102] Hu MC Shi M Zhang J Addo T Cho HJ Barker SL. Renal production, uptake, and handling of circulating αKlotho. J Am Soc Nephrol. (2016) 27:79–90. doi: 10.1681/asn.2014101030, PMID: 25977312 PMC4696570

[103] Lu X Hu MC. Klotho/FGF23 axis in chronic kidney disease and cardiovascular disease. Kidney Dis. (2017) 3:15–23. doi: 10.1159/000452880, PMID: 28785560 PMC5527179

[104] Zheng S Chen Y Zheng Y Zhou Z Li Z. Correlation of serum levels of fibroblast growth factor 23 and klotho protein levels with bone mineral density in maintenance hemodialysis patients. Eur J Med Res. (2018) 23:18. doi: 10.1186/s40001-018-0315-z, PMID: 29665846 PMC5905149

[105] Liu Z Zhou H Chen X Chen H Wang Y Wang T. Relationship between cFGF23/Klotho ratio and phosphate levels in patients with chronic kidney disease. Int Urol Nephrol. (2019) 51:503–7. doi: 10.1007/s11255-019-02079-4, PMID: 30689182

[106] Arroyo E Troutman AD Moorthi RN Avin KG Coggan AR Lim K. Klotho: An emerging factor with ergogenic potential. Front Rehabil Sci. (2021) 2:807123. doi: 10.3389/fresc.2021.807123, PMID: 36188832 PMC9397700

[107] Ix JH Shlipak MG. The promise of tubule biomarkers in kidney disease: a review. Am J Kidney Dis. (2021) 78:719–27. doi: 10.1053/j.ajkd.2021.03.026, PMID: 34051308 PMC8545710

[108] Al-Naimi MS Rasheed HA Hussien NR Al-Kuraishy HM Al-Gareeb AI. Nephrotoxicity: role and significance of renal biomarkers in the early detection of acute renal injury. J Adv Pharm Technol Res. (2019) 10:95–9. doi: 10.4103/japtr.JAPTR_336_18, PMID: 31334089 PMC6621352

[109] Lim WH Lewis JR Wong G Teo R Lim EM Byrnes E. Plasma neutrophil gelatinase-associated lipocalin and kidney function decline and kidney disease-related clinical events in older women. Am J Nephrol. (2015) 41:156–64. doi: 10.1159/000380831, PMID: 25824561

[110] Jungbauer CG Uecer E Stadler S Birner C Buchner S Maier LS. N-acteyl-ß-D-glucosaminidase and kidney injury molecule-1: new predictors for long-term progression of chronic kidney disease in patients with heart failure. Nephrology. (2016) 21:490–8. doi: 10.1111/nep.12632, PMID: 26422793

[111] Alderson HV Ritchie JP Pagano S Middleton RJ Pruijm M Vuilleumier N. The associations of blood kidney injury Molecule-1 and neutrophil gelatinase-associated lipocalin with progression from CKD to ESRD. Clin J Am Soc Nephrol. (2016) 11:2141–9. doi: 10.2215/cjn.02670316, PMID: 27852662 PMC5142061

[112] Hasegawa M Ishii J Kitagawa F Takahashi H Sugiyama K Tada M. Plasma neutrophil gelatinase-associated lipocalin as a predictor of cardiovascular events in patients with chronic kidney disease. Biomed Res Int. (2016) 2016:8761475–7. doi: 10.1155/2016/8761475, PMID: 27051671 PMC4808666

[113] Matsui K Kamijo-Ikemori A Imai N Sugaya T Yasuda T Tatsunami S. Clinical significance of urinary liver-type fatty acid-binding protein as a predictor of ESRD and CVD in patients with CKD. Clin Exp Nephrol. (2016) 20:195–203. doi: 10.1007/s10157-015-1144-9, PMID: 26189083

[114] Khatir DS Bendtsen MD Birn H Nørregaard R Ivarsen P Jespersen B. Urine liver fatty acid binding protein and chronic kidney disease progression. Scand J Clin Lab Invest. (2017) 77:549–54. doi: 10.1080/00365513.2017.1355561, PMID: 28745927

[115] Moriya H Mochida Y Ishioka K Oka M Maesato K Hidaka S. Plasma neutrophil gelatinase-associated lipocalin (NGAL) is an indicator of interstitial damage and a predictor of kidney function worsening of chronic kidney disease in the early stage: a pilot study. Clin Exp Nephrol. (2017) 21:1053–9. doi: 10.1007/s10157-017-1402-0 28397074

[116] Lobato GR Lobato MR Thomé FS Veronese FV. Performance of urinary kidney injury molecule-1, neutrophil gelatinase-associated lipocalin, and N-acetyl-β-D-glucosaminidase to predict chronic kidney disease progression and adverse outcomes. Braz J Med Biol Res. (2017) 50:e6106. doi: 10.1590/1414-431x20176106, PMID: 28380198 PMC5423741

[117] Zhang WR Craven TE Malhotra R Cheung AK Chonchol M Drawz P. Kidney damage biomarkers and incident chronic kidney disease during blood pressure reduction: a case-control study. Ann Intern Med. (2018) 169:610–8. doi: 10.7326/m18-1037, PMID: 30357395 PMC6953744

[118] Seibert FS Sitz M Passfall J Haesner M Laschinski P Buhl M. Prognostic value of urinary calprotectin, NGAL and KIM-1 in chronic kidney disease. Kidney Blood Press Res. (2018) 43:1255–62. doi: 10.1159/000492407, PMID: 30078006

[119] Żyłka A Dumnicka P Kuśnierz-Cabala B Gala-Błądzińska A Ceranowicz P Kucharz J. Markers of glomerular and tubular damage in the early stage of kidney disease in type 2 diabetic patients. Mediat Inflamm. (2018) 2018:7659243–12. doi: 10.1155/2018/7659243, PMID: 30158836 PMC6109534

[120] Steubl D Block M Herbst V Nockher WA Schlumberger W Kemmner S. Urinary uromodulin independently predicts end-stage renal disease and rapid kidney function decline in a cohort of chronic kidney disease patients. Medicine. (2019) 98:e15808. doi: 10.1097/md.0000000000015808, PMID: 31124979 PMC6571211

[121] Steubl D Buzkova P Garimella PS Ix JH Devarajan P Bennett MR. Association of serum uromodulin with ESKD and kidney function decline in the elderly: the cardiovascular health study. Am J Kidney Dis. (2019) 74:501–9. doi: 10.1053/j.ajkd.2019.02.024, PMID: 31128770 PMC7188359

[122] Malhotra R Katz R Jotwani V Ambrosius WT Raphael KL Haley W. Urine markers of kidney tubule cell injury and kidney function decline in SPRINT trial participants with CKD. Clin J Am Soc Nephrol. (2020) 15:349–58. doi: 10.2215/cjn.02780319, PMID: 32111704 PMC7057300

[123] Schulz CA Engström G Nilsson J Almgren P Petkovic M Christensson A. Plasma kidney injury molecule-1 (p-KIM-1) levels and deterioration of kidney function over 16 years. Nephrol Dial Transplant. (2020) 35:265–73. doi: 10.1093/ndt/gfy382, PMID: 30629206 PMC7049260

[124] Chen TK Coca SG Thiessen-Philbrook HR Heerspink HJL Obeid W Ix JH. Urinary biomarkers of tubular health and risk for kidney function decline or mortality in diabetes. Am J Nephrol. (2022) 53:775–85. doi: 10.1159/000528918, PMID: 36630924 PMC10006337

[125] Puthumana J Thiessen-Philbrook H Xu L Coca SG Garg AX Himmelfarb J. Biomarkers of inflammation and repair in kidney disease progression. J Clin Invest. (2021) 131:e139927. doi: 10.1172/jci139927, PMID: 33290282 PMC7843225

[126] Vasquez-Rios G Katz R Levitan EB Cushman M Parikh CR Kimmel PL. Urinary biomarkers of kidney tubule health and mortality in persons with CKD and diabetes mellitus. Kidney360. (2023) 4:e1257–64. doi: 10.34067/kid.0000000000000226 37533144 PMC10547219

[127] Flower DR. The lipocalin protein family: structure and function. Biochem J. (1996) 318:1–14. doi: 10.1042/bj3180001, PMID: 8761444 PMC1217580

[128] Mishra J Dent C Tarabishi R Mitsnefes MM Ma Q Kelly C. Neutrophil gelatinase-associated lipocalin (NGAL) as a biomarker for acute renal injury after cardiac surgery. Lancet. (2005) 365:1231–8. doi: 10.1016/s0140-6736(05)74811-x 15811456

[129] Yuen PS Jo SK Holly MK Hu X Star RA. Ischemic and nephrotoxic acute renal failure are distinguished by their broad transcriptomic responses. Physiol Genomics. (2006) 25:375–86. doi: 10.1152/physiolgenomics.00223.2005, PMID: 16507785 PMC1502395

[130] Bjornstad P Pyle L Cherney DZI Johnson RJ Sippl R Wong R. Plasma biomarkers improve prediction of diabetic kidney disease in adults with type 1 diabetes over a 12-year follow-up: CACTI study. Nephrol Dial Transplant. (2018) 33:1189–96. doi: 10.1093/ndt/gfx255, PMID: 28992280 PMC6030887

[131] Nickolas TL Forster CS Sise ME Barasch N Valle DSD Viltard M. NGAL (Lcn2) monomer is associated with tubulointerstitial damage in chronic kidney disease. Kidney Int. (2012) 82:718–22. doi: 10.1038/ki.2012.195 22695331 PMC3519389

[132] Hasegawa M Ishii J Kitagawa F Takahashi K Hayashi H Koide S. Urinary neutrophil gelatinase-associated lipocalin as a predictor of cardiovascular events in patients with chronic kidney disease. Heart Vessel. (2015) 30:81–8. doi: 10.1007/s00380-013-0454-7, PMID: 24378882

[133] Devarajan P. Neutrophil gelatinase-associated lipocalin (NGAL): a new marker of kidney disease. Scand J Clin Lab Invest Suppl. (2008) 241:89–94. doi: 10.1080/00365510802150158, PMID: 18569973 PMC2528839

[134] Huo W Zhang K Nie Z Li Q Jin F. Kidney injury molecule-1 (KIM-1): a novel kidney-specific injury molecule playing potential double-edged functions in kidney injury. Transplant Rev. (2010) 24:143–6. doi: 10.1016/j.trre.2010.02.002, PMID: 20447817

[135] Novak R Salai G Hrkac S Vojtusek IK Grgurevic L. Revisiting the role of NAG across the continuum of kidney disease. Bioengineering. (2023) 10:444. doi: 10.3390/bioengineering10040444, PMID: 37106631 PMC10136202

[136] Yin C Wang N. Kidney injury molecule-1 in kidney disease. Ren Fail. (2016) 38:1567–73. doi: 10.1080/0886022x.2016.1193816 27758121

[137] Sabbisetti VS Waikar SS Antoine DJ Smiles A Wang C Ravisankar A. Blood kidney injury molecule-1 is a biomarker of acute and chronic kidney injury and predicts progression to ESRD in type I diabetes. J Am Soc Nephrol. (2014) 25:2177–86. doi: 10.1681/asn.2013070758, PMID: 24904085 PMC4178434

[138] Wajda J Dumnicka P Kolber W Sporek M Maziarz B Ceranowicz P. The marker of tubular injury, kidney injury molecule-1 (KIM-1), in acute kidney injury complicating acute pancreatitis: a preliminary study. J Clin Med. (2020) 9:1463. doi: 10.3390/jcm9051463, PMID: 32414176 PMC7290845

[139] Mizdrak M Kumrić M Kurir TT Božić J. Emerging biomarkers for early detection of chronic kidney disease. J Pers Med. (2022) 12:548. doi: 10.3390/jpm12040548, PMID: 35455664 PMC9025702

[140] Xu Y Xie Y Shao X Ni Z Mou S. L-FABP: a novel biomarker of kidney disease. Clin Chim Acta. (2015) 445:85–90. doi: 10.1016/j.cca.2015.03.017 25797895

[141] Yamamoto T Noiri E Ono Y Doi K Negishi K Kamijo A. Renal L-type fatty acid--binding protein in acute ischemic injury. J Am Soc Nephrol. (2007) 18:2894–902. doi: 10.1681/asn.2007010097, PMID: 17942962

[142] Parikh CR Thiessen-Philbrook H Garg AX Kadiyala D Shlipak MG Koyner JL. Performance of kidney injury molecule-1 and liver fatty acid-binding protein and combined biomarkers of AKI after cardiac surgery. Clin J Am Soc Nephrol. (2013) 8:1079–88. doi: 10.2215/cjn.10971012, PMID: 23599408 PMC3700701

[143] Portilla D Dent C Sugaya T Nagothu KK Kundi I Moore P. Liver fatty acid-binding protein as a biomarker of acute kidney injury after cardiac surgery. Kidney Int. (2008) 73:465–72. doi: 10.1038/sj.ki.5002721 18094680

[144] Kamijo A Sugaya T Hikawa A Yamanouchi M Hirata Y Ishimitsu T. Urinary liver-type fatty acid binding protein as a useful biomarker in chronic kidney disease. Mol Cell Biochem. (2006) 284:175–82. doi: 10.1007/s11010-005-9047-9 16532260

[145] Maeda Y Suzuki A Ishii J Sekiguchi-Ueda S Shibata M Yoshino Y. Level of urinary liver-type fatty acid-binding protein is associated with cardiac markers and electrocardiographic abnormalities in type-2 diabetes with chronic kidney disease stage G1 and G2. Heart Vessel. (2015) 30:362–8. doi: 10.1007/s00380-014-0489-4, PMID: 24626813

[146] Devuyst O Olinger E Rampoldi L. Uromodulin: from physiology to rare and complex kidney disorders. Nat Rev Nephrol. (2017) 13:525–44. doi: 10.1038/nrneph.2017.101, PMID: 28781372

[147] Schaeffer C Devuyst O Rampoldi L. Uromodulin: roles in health and disease. Annu Rev Physiol. (2021) 83:477–501. doi: 10.1146/annurev-physiol-031620-092817 33566673

[148] Scherberich JE Gruber R Nockher WA Christensen EI Schmitt H Herbst V. Serum uromodulin—a marker of kidney function and renal parenchymal integrity. Nephrol Dial Transplant. (2018) 33:284–95. doi: 10.1093/ndt/gfw422, PMID: 28206617 PMC5837243

[149] Fedak D Kuźniewski M Fugiel A Wieczorek-Surdacka E Przepiórkowska-Hoyer B Jasik P. Serum uromodulin concentrations correlate with glomerular filtration rate in patients with chronic kidney disease. Pol Arch Med Wewn. (2016) 126:995–1004. doi: 10.20452/pamw.3712, PMID: 27958261

[150] Delgado GE Kleber ME Scharnagl H Krämer BK März W Scherberich JE. Serum uromodulin and mortality risk in patients undergoing coronary angiography. J Am Soc Nephrol. (2017) 28:2201–10. doi: 10.1681/asn.2016111162, PMID: 28242751 PMC5491294

[151] Amdur RL Feldman HI Gupta J Yang W Kanetsky P Shlipak M. Inflammation and progression of CKD: the CRIC study. Clin J Am Soc Nephrol. (2016) 11:1546–56. doi: 10.2215/cjn.13121215, PMID: 27340285 PMC5012490

[152] Mihai S Codrici E Popescu ID Enciu AM Albulescu L Necula LG. Inflammation-related mechanisms in chronic kidney disease prediction, progression, and outcome. J Immunol Res. (2018) 2018:2180373–16. doi: 10.1155/2018/2180373, PMID: 30271792 PMC6146775

[153] Petreski T Piko N Ekart R Hojs R Bevc S. Review on inflammation markers in chronic kidney disease. Biomedicines. (2021) 9:182. doi: 10.3390/biomedicines9020182, PMID: 33670423 PMC7917900

[154] Kadatane SP Satariano M Massey M Mongan K Raina R. The role of inflammation in CKD. Cells. (2023) 12:1581. doi: 10.3390/cells12121581, PMID: 37371050 PMC10296717

[155] Huang R Fu P Ma L. Kidney fibrosis: from mechanisms to therapeutic medicines. Signal Transduct Target Ther. (2023) 8:129. doi: 10.1038/s41392-023-01379-7, PMID: 36932062 PMC10023808

[156] Sjöberg B Qureshi AR Heimbürger O Stenvinkel P Lind L Larsson A. Association between levels of pentraxin 3 and incidence of chronic kidney disease in the elderly. J Intern Med. (2016) 279:173–9. doi: 10.1111/joim.12411, PMID: 26355706 PMC4737281

[157] Sun J Axelsson J Machowska A Heimbürger O Bárány P Lindholm B. Biomarkers of cardiovascular disease and mortality risk in patients with advanced CKD. Clin J Am Soc Nephrol. (2016) 11:1163–72. doi: 10.2215/cjn.10441015, PMID: 27281698 PMC4934843

[158] Fernández-Juárez G Villacorta Perez J Luño Fernández JL Martinez-Martinez E Cachofeiro V Barrio Lucia V. High levels of circulating TNFR1 increase the risk of all-cause mortality and progression of renal disease in type 2 diabetic nephropathy. Nephrology. (2017) 22:354–60. doi: 10.1111/nep.12781, PMID: 27003829

[159] Gohda T Maruyama S Kamei N Yamaguchi S Shibata T Murakoshi M. Circulating TNF receptors 1 and 2 predict mortality in patients with end-stage renal disease undergoing dialysis. Sci Rep. (2017) 7:43520. doi: 10.1038/srep43520, PMID: 28256549 PMC5335256

[160] Nair V Robinson-Cohen C Smith MR Bellovich KA Bhat ZY Bobadilla M. Growth differentiation factor-15 and risk of CKD progression. J Am Soc Nephrol. (2017) 28:2233–40. doi: 10.1681/asn.2016080919, PMID: 28159780 PMC5491285

[161] Krzanowski M Krzanowska K Gajda M Dumnicka P Dziewierz A Woziwodzka K. Pentraxin 3 as a new indicator of cardiovascular-related death in patients with advanced chronic kidney disease. Pol Arch Intern Med. (2017) 127:170–7. doi: 10.20452/pamw.3944, PMID: 28377558

[162] Tuegel C Katz R Alam M Bhat Z Bellovich K de Boer I. GDF-15, galectin 3, soluble ST2, and risk of mortality and cardiovascular events in CKD. Am J Kidney Dis. (2018) 72:519–28. doi: 10.1053/j.ajkd.2018.03.025, PMID: 29866459 PMC6153047

[163] Frimodt-Møller M von Scholten BJ Reinhard H Jacobsen PK Hansen TW Persson FI. Growth differentiation factor-15 and fibroblast growth factor-23 are associated with mortality in type 2 diabetes - An observational follow-up study. PLoS One. (2018) 13:e0196634. doi: 10.1371/journal.pone.0196634, PMID: 29698460 PMC5919646

[164] Kamińska J Stopiński M Mucha K Jędrzejczak A Gołębiowski M Niewczas MA. IL 6 but not TNF is linked to coronary artery calcification in patients with chronic kidney disease. Cytokine. (2019) 120:9–14. doi: 10.1016/j.cyto.2019.04.002 30991230

[165] Valente MJ Rocha S Coimbra S Catarino C Rocha-Pereira P Bronze-da-Rocha E. Long pentraxin 3 as a broader biomarker for multiple risk factors in end-stage renal disease: association with all-cause mortality. Mediat Inflamm. (2019) 2019:3295725–12. doi: 10.1155/2019/3295725, PMID: 31316299 PMC6604294

[166] Vega A Sanchez-Niño MD Ortiz A Abad S Macías N Aragoncillo I. The new marker YKL-40, a molecule related to inflammation, is associated with cardiovascular events in stable haemodialysis patients. Clin Kidney J. (2020) 13:172–8. doi: 10.1093/ckj/sfz056, PMID: 32296521 PMC7147298

[167] Batra G Ghukasyan Lakic T Lindbäck J Held C White HD Stewart RAH. Interleukin 6 and cardiovascular outcomes in patients with chronic kidney disease and chronic coronary syndrome. JAMA Cardiol. (2021) 6:1440–5. doi: 10.1001/jamacardio.2021.3079, PMID: 34431970 PMC8387946

[168] Scurt FG Menne J Brandt S Bernhardt A Mertens PR Haller H. Monocyte chemoattractant protein-1 predicts the development of diabetic nephropathy. Diabetes Metab Res Rev. (2022) 38:e3497. doi: 10.1002/dmrr.3497 34541760

[169] Waijer SW Sen T Arnott C Neal B Kosterink JGW Mahaffey KW. Association between TNF receptors and KIM-1 with kidney outcomes in early-stage diabetic kidney disease. Clin J Am Soc Nephrol. (2022) 17:251–9. doi: 10.2215/cjn.08780621, PMID: 34876454 PMC8823939

[170] Li X Qureshi AR Suliman ME Heimburger O Barany P Stenvinkel P. Interleukin-6-to-albumin ratio as a superior predictor of mortality in end-stage kidney disease patients. Am J Nephrol. (2023) 54:268–74. doi: 10.1159/000531191, PMID: 37231796 PMC10623391

[171] Su H Lei CT Zhang C. Interleukin-6 signaling pathway and its role in kidney disease: an update. Front Immunol. (2017) 8:405. doi: 10.3389/fimmu.2017.00405, PMID: 28484449 PMC5399081

[172] Romanova Y Laikov A Markelova M Khadiullina R Makseev A Hasanova M. Proteomic analysis of human serum from patients with chronic kidney disease. Biomol Ther. (2020) 10:257. doi: 10.3390/biom10020257, PMID: 32046176 PMC7072325

[173] Lin Z Li H He C Yang M Chen H Yang X. Metabolomic biomarkers for the diagnosis and post-transplant outcomes of AFP negative hepatocellular carcinoma. Front Oncol. (2023) 13:1072775. doi: 10.3389/fonc.2023.1072775, PMID: 36845695 PMC9947281

[174] Durlacher-Betzer K Hassan A Levi R Axelrod J Silver J Naveh-Many T. Interleukin-6 contributes to the increase in fibroblast growth factor 23 expression in acute and chronic kidney disease. Kidney Int. (2018) 94:315–25. doi: 10.1016/j.kint.2018.02.026, PMID: 29861060

[175] Hirooka Y Nozaki Y. Interleukin-18 in inflammatory kidney disease. Front Med. (2021) 8:639103. doi: 10.3389/fmed.2021.639103, PMID: 33732720 PMC7956987

[176] Milas O Gadalean F Vlad A Dumitrascu V Velciov S Gluhovschi C. Pro-inflammatory cytokines are associated with podocyte damage and proximal tubular dysfunction in the early stage of diabetic kidney disease in type 2 diabetes mellitus patients. J Diabetes Complicat. (2020) 34:107479. doi: 10.1016/j.jdiacomp.2019.107479, PMID: 31806428

[177] Luan J Fu J Jiao C Hao X Feng Z Zhu L. IL-18 deficiency ameliorates the progression from AKI to CKD. Cell Death Dis. (2022) 13:957. doi: 10.1038/s41419-022-05394-4, PMID: 36379914 PMC9666542

[178] Lousa I Reis F Santos-Silva A Belo L. The signaling pathway of TNF receptors: linking animal models of renal disease to human CKD. Int J Mol Sci. (2022) 23:3284. doi: 10.3390/ijms23063284, PMID: 35328704 PMC8950598

[179] Bae E Cha RH Kim YC An JN Kim DK Yoo KD. Circulating TNF receptors predict cardiovascular disease in patients with chronic kidney disease. Medicine. (2017) 96:e6666. doi: 10.1097/md.0000000000006666, PMID: 28489742 PMC5428576

[180] Murakoshi M Gohda T Suzuki Y. Circulating tumor necrosis factor receptors: a potential biomarker for the progression of diabetic kidney disease. Int J Mol Sci. (2020) 21:1957. doi: 10.3390/ijms21061957, PMID: 32183005 PMC7139523

[181] Gohda T Niewczas MA Ficociello LH Walker WH Skupien J Rosetti F. Circulating TNF receptors 1 and 2 predict stage 3 CKD in type 1 diabetes. J Am Soc Nephrol. (2012) 23:516–24. doi: 10.1681/asn.2011060628, PMID: 22266664 PMC3294299

[182] Haller H Bertram A Nadrowitz F Menne J. Monocyte chemoattractant protein-1 and the kidney. Curr Opin Nephrol Hypertens. (2016) 25:42–9. doi: 10.1097/mnh.0000000000000186 26625862

[183] Ho JE Hwang SJ Wollert KC Larson MG Cheng S Kempf T. Biomarkers of cardiovascular stress and incident chronic kidney disease. Clin Chem. (2013) 59:1613–20. doi: 10.1373/clinchem.2013.205716, PMID: 23873716 PMC3972213

[184] Mirna M Topf A Wernly B Rezar R Paar V Jung C. Novel biomarkers in patients with chronic kidney disease: an analysis of patients enrolled in the GCKD-study. J Clin Med. (2020) 9:886. doi: 10.3390/jcm9030886, PMID: 32213894 PMC7141541

[185] Bansal N Zelnick L Shlipak MG Anderson A Christenson R Deo R. Cardiac and stress biomarkers and chronic kidney disease progression: the CRIC study. Clin Chem. (2019) 65:1448–57. doi: 10.1373/clinchem.2019.305797, PMID: 31578216 PMC6927328

[186] Wei C Spear R Hahm E Reiser J. suPAR, a circulating kidney disease factor. Front Med. (2021) 8:745838. doi: 10.3389/fmed.2021.745838, PMID: 34692736 PMC8526732

[187] Singh S Anshita D Ravichandiran V. MCP-1: function, regulation, and involvement in disease. Int Immunopharmacol. (2021) 101:107598. doi: 10.1016/j.intimp.2021.107598, PMID: 34233864 PMC8135227

[188] BGS. Available at: https://www.bgs.org.uk/sites/default/files/content/resources/files/2019-02-08/BGS%20Toolkit%20-%20FINAL%20FOR%20WEB_0.pdf (Accessed May 13, 2022).

[189] Pavasini R Guralnik J Brown JC di Bari M Cesari M Landi F. Short physical performance battery and all-cause mortality: systematic review and meta-analysis. BMC Med. (2016) 14:215. doi: 10.1186/s12916-016-0763-7, PMID: 28003033 PMC5178082

[190] Corsonello A Pedone C Bandinelli S Ferrucci L Antonelli Incalzi R. Predicting survival of older community-dwelling individuals according to five estimated glomerular filtration rate equations: the InChianti study. Geriatr Gerontol Int. (2018) 18:607–14. doi: 10.1111/ggi.13225, PMID: 29356245 PMC5891358

[191] Vatanabe IP Pedroso RV Teles RHG Ribeiro JC Manzine PR Pott-Junior H. A systematic review and meta-analysis on cognitive frailty in community-dwelling older adults: risk and associated factors. Aging Ment Health. (2021) 26:464–76. doi: 10.1080/13607863.2021.1884844, PMID: 33612030

[192] Kiesswetter E Pohlhausen S Uhlig K Diekmann R Lesser S Uter W. Prognostic differences of the mini nutritional assessment short form and long form in relation to 1-year functional decline and mortality in community-dwelling older adults receiving home care. J Am Geriatr Soc. (2014) 62:512–7. doi: 10.1111/jgs.12683, PMID: 24611678

[193] Lattanzio F Corsonello A Montesanto A Abbatecola AM Lofaro D Passarino G. Disentangling the impact of chronic kidney disease, anemia, and mobility limitation on mortality in older patients discharged from hospital. J Gerontol A Biol Sci Med Sci. (2015) 70:1120–7. doi: 10.1093/gerona/glv068, PMID: 25991829

[194] Soraci L Corica F Corsonello A Remelli F Abete P Bellelli G. Prognostic interplay of kidney function with sarcopenia, anemia, disability and cognitive impairment. The GLISTEN study. Eur J Intern Med. (2021) 93:57–63. doi: 10.1016/j.ejim.2021.06.031, PMID: 34253448

[195] Weiss JW Boyd CM. Managing complexity in older patients with CKD. Clin J Am Soc Nephrol. (2017) 12:559–61. doi: 10.2215/cjn.02340317, PMID: 28389528 PMC5383380

[196] Wang XH Mitch WE Price SR. Pathophysiological mechanisms leading to muscle loss in chronic kidney disease. Nat Rev Nephrol. (2022) 18:138–52. doi: 10.1038/s41581-021-00498-0, PMID: 34750550

[197] Rong YD Bian AL Hu HY Ma Y Zhou XZ. Study on relationship between elderly sarcopenia and inflammatory cytokine IL-6, anti-inflammatory cytokine IL-10. BMC Geriatr. (2018) 18:308. doi: 10.1186/s12877-018-1007-9, PMID: 30541467 PMC6292155

[198] Franceschi C Bonafè M Valensin S Olivieri F De Luca M Ottaviani E. An evolutionary perspective on immunosenescence. Ann N Y Acad Sci. (2000) 908:244–54. doi: 10.1111/j.1749-6632.2000.tb06651.x, PMID: 10911963

[199] Franceschi C Garagnani P Parini P Giuliani C Santoro A. Inflammaging: a new immune-metabolic viewpoint for age-related diseases. Nat Rev Endocrinol. (2018) 14:576–90. doi: 10.1038/s41574-018-0059-4, PMID: 30046148

[200] Huang Y Wang B Hassounah F Price SR Klein J Mohamed TMA. The impact of senescence on muscle wasting in chronic kidney disease. J Cachexia Sarcopenia Muscle. (2023) 14:126–41. doi: 10.1002/jcsm.13112, PMID: 36351875 PMC9891952

[201] Huang Z Zhong L Zhu J Xu H Ma W Zhang L. Inhibition of IL-6/JAK/STAT3 pathway rescues denervation-induced skeletal muscle atrophy. Ann Transl Med. (2020) 8:1681. doi: 10.21037/atm-20-7269, PMID: 33490193 PMC7812230

[202] Zhang N Zheng Q Wang Y Lin J Wang H Liu R. Renoprotective effect of the recombinant anti-IL-6R fusion proteins by inhibiting JAK2/STAT3 signaling pathway in diabetic nephropathy. Front Pharmacol. (2021) 12:681424. doi: 10.3389/fphar.2021.681424, PMID: 34054555 PMC8155588

[203] Liu T Gao H Zhang Y Wang S Lu M Dai X. Apigenin ameliorates hyperuricemia and renal injury through regulation of uric acid metabolism and JAK2/STAT3 signaling pathway. Pharmaceuticals. (2022) 15:1442. doi: 10.3390/ph15111442, PMID: 36422572 PMC9697024

[204] He Y Xie W Li H Jin H Zhang Y Li Y. Cellular senescence in sarcopenia: possible mechanisms and therapeutic potential. Front Cell Dev Biol. (2021) 9:793088. doi: 10.3389/fcell.2021.793088, PMID: 35083219 PMC8784872

[205] Clemens Z Sivakumar S Pius A Sahu A Shinde S Mamiya H. The biphasic and age-dependent impact of klotho on hallmarks of aging and skeletal muscle function. eLife. (2021) 10:e61138. doi: 10.7554/eLife.61138, PMID: 33876724 PMC8118657

[206] Dozio E Vettoretti S Lungarella G Messa P Corsi Romanelli MM. Sarcopenia in chronic kidney disease: focus on advanced glycation end products as mediators and markers of oxidative stress. Biomedicines. (2021) 9:405. doi: 10.3390/biomedicines9040405, PMID: 33918767 PMC8068965

[207] Sabatino A Cuppari L Stenvinkel P Lindholm B Avesani CM. Sarcopenia in chronic kidney disease: what have we learned so far? J Nephrol. (2021) 34:1347–72. doi: 10.1007/s40620-020-00840-y, PMID: 32876940 PMC8357704

[208] Bhat M Kalam R Qadri SSYH Madabushi S Ismail A. Vitamin D deficiency-induced muscle wasting occurs through the ubiquitin proteasome pathway and is partially corrected by calcium in male rats. Endocrinology. (2013) 154:4018–29. doi: 10.1210/en.2013-1369, PMID: 23928374

[209] Zhang F Wang H Bai Y Zhang Y Huang L Zhang H. Prevalence of physical frailty and impact on survival in patients with chronic kidney disease: a systematic review and meta-analysis. BMC Nephrol. (2023) 24:258. doi: 10.1186/s12882-023-03303-1, PMID: 37661257 PMC10476333

[210] Mielke N Schneider A Barghouth MH Ebert N van der Giet M Huscher D. Association of kidney function and albuminuria with frailty worsening and death in very old adults. Age Ageing. (2023) 52:afad063. doi: 10.1093/ageing/afad063, PMID: 37192504

[211] Vanholder R Van Laecke S Glorieux G. What is new in uremic toxicity? Pediatr Nephrol. (2008) 23:1211–21. doi: 10.1007/s00467-008-0762-9, PMID: 18324423 PMC2441592

[212] Brouns R De Deyn PP. Neurological complications in renal failure: a review. Clin Neurol Neurosurg. (2004) 107:1–16. doi: 10.1016/j.clineuro.2004.07.012 15567546

[213] Watanabe K Watanabe T Nakayama M. Cerebro-renal interactions: impact of uremic toxins on cognitive function. Neurotoxicology. (2014) 44:184–93. doi: 10.1016/j.neuro.2014.06.014, PMID: 25003961

[214] de Donato A Buonincontri V Borriello G Martinelli G Mone P. The dopamine system: insights between kidney and brain. Kidney Blood Press Res. (2022) 47:493–505. doi: 10.1159/000522132 35378538

[215] Iwata Y Nakade Y Kinoshita M Sabit H Nakajima R Furuichi K. Intra-brain and plasma levels of L-serine are associated with cognitive status in patients with chronic kidney disease. Kidney Dis. (2023) 9:118–30. doi: 10.1159/000527798, PMID: 37065608 PMC10090982

[216] Natale G Calabrese V Marino G Campanelli F Urciuolo F de Iure A. Effects of uremic toxins on hippocampal synaptic transmission: implication for neurodegeneration in chronic kidney disease. Cell Death Discov. (2021) 7:295. doi: 10.1038/s41420-021-00685-9, PMID: 34657122 PMC8520534

[217] Li LC Chen WY Chen JB Lee WC Chang CC Tzeng HT. The AST-120 recovers uremic toxin-induced cognitive deficit via NLRP3 inflammasome pathway in astrocytes and microglia. Biomedicines. (2021) 9:1252. doi: 10.3390/biomedicines9091252, PMID: 34572437 PMC8467651

[218] Lauretani F Meschi T Ticinesi A Maggio M. “Brain-muscle loop” in the fragility of older persons: from pathophysiology to new organizing models. Aging Clin Exp Res. (2017) 29:1305–11. doi: 10.1007/s40520-017-0729-4 28233284

[219] Zhou Y Hellberg M Svensson P Höglund P Clyne N. Sarcopenia and relationships between muscle mass, measured glomerular filtration rate and physical function in patients with chronic kidney disease stages 3–5. Nephrol Dial Transplant. (2018) 33:342–8. doi: 10.1093/ndt/gfw466, PMID: 28340152

[220] Souza VA Oliveira D Barbosa SR Corrêa JOA Colugnati FAB Mansur HN. Sarcopenia in patients with chronic kidney disease not yet on dialysis: analysis of the prevalence and associated factors. PLoS One. (2017) 12:e0176230. doi: 10.1371/journal.pone.0176230, PMID: 28448584 PMC5407780

[221] Foley RN Wang C Ishani A Collins AJ Murray AM. Kidney function and sarcopenia in the United States general population: NHANES III. Am J Nephrol. (2007) 27:279–86. doi: 10.1159/000101827, PMID: 17440263

[222] Moreno-Gonzalez R Corbella X Mattace-Raso F Tap L Sieber C Freiberger E. Prevalence of sarcopenia in community-dwelling older adults using the updated EWGSOP2 definition according to kidney function and albuminuria: the screening for CKD among older people across Europe (SCOPE) study. BMC Geriatr. (2020) 20:327. doi: 10.1186/s12877-020-01700-x, PMID: 33008317 PMC7531109

[223] Kim JK Choi SR Choi MJ Kim SG Lee YK Noh JW. Prevalence of and factors associated with sarcopenia in elderly patients with end-stage renal disease. Clin Nutr. (2014) 33:64–8. doi: 10.1016/j.clnu.2013.04.002 23631844

[224] Antuña E Cachán-Vega C Bermejo-Millo JC Potes Y Caballero B Vega-Naredo I. Inflammaging: implications in sarcopenia. Int J Mol Sci. (2022) 23:15039. doi: 10.3390/ijms232315039, PMID: 36499366 PMC9740553

[225] Han E Lee YH Kim G Kim SR Lee BW Kang ES. Sarcopenia is associated with albuminuria independently of hypertension and diabetes: KNHANES 2008–2011. Metabolism. (2016) 65:1531–40. doi: 10.1016/j.metabol.2016.07.003, PMID: 27621188

[226] Bouchi R Fukuda T Takeuchi T Minami I Yoshimoto T Ogawa Y. Sarcopenia is associated with incident albuminuria in patients with type 2 diabetes: a retrospective observational study. J Diabetes Investig. (2017) 8:783–7. doi: 10.1111/jdi.12636, PMID: 28130832 PMC5668516

[227] Avesani CM de Abreu AM Ribeiro HS Brismar TB Stenvinkel P Sabatino A. Muscle fat infiltration in chronic kidney disease: a marker related to muscle quality, muscle strength and sarcopenia. J Nephrol. (2023) 36:895–910. doi: 10.1007/s40620-022-01553-0, PMID: 36719556 PMC10090035

[228] Huang YM Chen WM Chen M Shia BC Wu SY. Sarcopenia is an independent risk factor for severe diabetic nephropathy in type 2 diabetes: a long-term follow-up propensity score-matched diabetes cohort study. J Clin Med. (2022) 11:2992. doi: 10.3390/jcm11112992, PMID: 35683381 PMC9181390

[229] Ribeiro HS Neri SGR Oliveira JS Bennett PN Viana JL Lima RM. Association between sarcopenia and clinical outcomes in chronic kidney disease patients: a systematic review and meta-analysis. Clin Nutr. (2022) 41:1131–40. doi: 10.1016/j.clnu.2022.03.025, PMID: 35430544

[230] Wathanavasin W Banjongjit A Avihingsanon Y Praditpornsilpa K Tungsanga K Eiam-Ong S. Prevalence of sarcopenia and its impact on cardiovascular events and mortality among dialysis patients: a systematic review and Meta-analysis. Nutrients. (2022) 14:4077. doi: 10.3390/nu14194077, PMID: 36235729 PMC9572026

[231] Corsonello A Roller-Wirnsberger R di Rosa M Fabbietti P Wirnsberger G Kostka T. Estimated glomerular filtration rate and functional status among older people: a systematic review. Eur J Intern Med. (2018) 56:39–48. doi: 10.1016/j.ejim.2018.05.030, PMID: 29936073

[232] Corsonello A Lattanzio F. Unveiling the geriatric dimensions of chronic kidney disease. Age Ageing. (2023) 52:afad117. doi: 10.1093/ageing/afad117, PMID: 37389556

[233] Fulinara CP Huynh A Goldwater D Abdalla B Schaenman J. Frailty and age-associated assessments associated with chronic kidney disease and transplantation outcomes. J Transp Secur. (2023) 2023:1510259–16. doi: 10.1155/2023/1510259, PMID: 37038595 PMC10082678

[234] Nixon AC Bampouras TM Pendleton N Woywodt A Mitra S Dhaygude A. Frailty and chronic kidney disease: current evidence and continuing uncertainties. Clin Kidney J. (2018) 11:236–45. doi: 10.1093/ckj/sfx134, PMID: 29644065 PMC5888002

[235] Kennard AL Rainsford S Glasgow NJ Talaulikar GS. Use of frailty assessment instruments in nephrology populations: a scoping review. BMC Geriatr. (2023) 23:449. doi: 10.1186/s12877-023-04101-y, PMID: 37479978 PMC10360289

[236] Lorenz EC Kennedy CC Rule AD LeBrasseur NK Kirkland JL Hickson LTJ. Frailty in CKD and transplantation. Kidney Int Rep. (2021) 6:2270–80. doi: 10.1016/j.ekir.2021.05.025, PMID: 34514190 PMC8418946

[237] Kennard A Glasgow N Rainsford S Talaulikar G. Frailty in chronic kidney disease: challenges in nephrology practice. A review of current literature. Int Med J. (2023) 53:465–72. doi: 10.1111/imj.15759, PMID: 35353436

[238] Walker SR Gill K Macdonald K Komenda P Rigatto C Sood MM. Association of frailty and physical function in patients with non-dialysis CKD: a systematic review. BMC Nephrol. (2013) 14:228. doi: 10.1186/1471-2369-14-228, PMID: 24148266 PMC4016413

[239] Mei F Gao Q Chen F Zhao L Shang Y Hu K. Frailty as a predictor of negative health outcomes in chronic kidney disease: a systematic review and meta-analysis. J Am Med Dir Assoc. (2021) 22:535–543.e7. doi: 10.1016/j.jamda.2020.09.033, PMID: 33218914

[240] Zhang H Hao M Li Y Jiang X Wang M Chen J. Glomerular filtration rate by different measures and albuminuria are associated with risk of frailty: the Rugao longitudinal ageing study. Aging Clin Exp Res. (2022) 34:2703–11. doi: 10.1007/s40520-022-02245-2, PMID: 36260213

[241] Etgen T Chonchol M Förstl H Sander D. Chronic kidney disease and cognitive impairment: a systematic review and meta-analysis. Am J Nephrol. (2012) 35:474–82. doi: 10.1159/000338135 22555151

[242] Tap L Corsonello A Formiga F Moreno-Gonzalez R Ärnlöv J Carlsson AC. Is kidney function associated with cognition and mood in late life?: The screening for CKD among older people across Europe (SCOPE) study. BMC Geriatr. (2020) 20:297. doi: 10.1186/s12877-020-01707-4, PMID: 33008359 PMC7531080

[243] Chang J Hou W Li Y Li S Zhao K Wang Y. Prevalence and associated factors of cognitive frailty in older patients with chronic kidney disease: a cross-sectional study. BMC Geriatr. (2022) 22:681. doi: 10.1186/s12877-022-03366-z, PMID: 35978304 PMC9386941

[244] Luo B Luo Z Zhang X Xu M Shi C. Status of cognitive frailty in elderly patients with chronic kidney disease and construction of a risk prediction model: a cross-sectional study. BMJ Open. (2022) 12:e060633. doi: 10.1136/bmjopen-2021-060633, PMID: 36572488 PMC9806025

[245] Scheppach JB Wu A Gottesman RF Mosley TH Arsiwala-Scheppach LT Knopman DS. Association of kidney function measures with signs of neurodegeneration and small vessel disease on brain magnetic resonance imaging: the atherosclerosis risk in communities (ARIC) study. Am J Kidney Dis. (2023) 81:261–269.e1. doi: 10.1053/j.ajkd.2022.07.013, PMID: 36179945 PMC9974563

[246] Feng X Hou N Chen Z Liu J Li X Sun X. Secular trends of epidemiologic patterns of chronic kidney disease over three decades: an updated analysis of the Global Burden of Disease Study 2019. BMJ Open. (2023) 13:e064540. doi: 10.1136/bmjopen-2022-064540, PMID: 36931681 PMC10030786

[247] Ke C Liang J Liu M Liu S Wang C. Burden of chronic kidney disease and its risk-attributable burden in 137 low-and middle-income countries, 1990-2019: results from the global burden of disease study 2019. BMC Nephrol. (2022) 23:17. doi: 10.1186/s12882-021-02597-3, PMID: 34986789 PMC8727977

[248] Delanaye P Jager KJ Bökenkamp A Christensson A Dubourg L Eriksen BO. CKD: a call for an age-adapted definition. J Am Soc Nephrol. (2019) 30:1785–805. doi: 10.1681/asn.2019030238, PMID: 31506289 PMC6779354

[249] Glassock R Delanaye P El Nahas M. An age-calibrated classification of chronic kidney disease. JAMA Cardiol. (2015) 314:559–60. doi: 10.1001/jama.2015.6731, PMID: 26023760

[250] Levey AS Inker LA Coresh J. Chronic kidney disease in older people. JAMA. (2015) 314:557–8. doi: 10.1001/jama.2015.6753 26023868

[251] Writing Group for the CKD Prognosis Consortium. Estimated glomerular filtration rate, albuminuria, and adverse outcomes: an individual-participant data meta-analysis. JAMA. (2023) 330:1266–77. doi: 10.1001/jama.2023.17002, PMID: 37787795 PMC10548311

